# Tumour necrosis factor: a cytokine with multiple biological activities.

**DOI:** 10.1038/bjc.1990.78

**Published:** 1990-03

**Authors:** G. Semenzato

**Affiliations:** Istituto di Medicina Clinica dell'Università di Padova, Clinica Medica 1, Italy.


Br. J. Cancer (1990), 61, 354-361 © Macmillan Press Ltd., 1990

REVIEW

### Tumour necrosis factor: a cytokine with multiple biological activities

G. Semenzato

*Istituto di Medicina Clinica dell' Università di Padova, Clinica Medica 1, Via Giustiniani 2, Padova, Italy.*

Recently accumulated data provided evidence that cytokines modulate and/or mediate many essential biological processes, in particular those concerned with cell growth and differentiation. These molecules are also mandatory for the regulation of numerous inflammatory and physiological states by displaying a broad range of biological properties. The term biological response modifier that has been applied to the substances belonging to this group extensively encompasses the wide spectrum of activities covered by these molecules. A single cytokine may have multiple effects both *in vivo* and *in vitro*, with these properties often overlapping each other and the final result being the sum of the actions of different factors. One of these molecules with pleiomorphic functions is tumour necrosis factor (TNF).

The name assigned to TNF is descriptive of the historical activity of this cytokine and nowadays does not reflect the true spectrum of its biological activity. Lymphotoxin was initially discovered as a cytotoxic factor produced by T cells (Granger & Williams, 1968) and then the term TNF was introduced to describe a serum protein produced after bacterial infections which is capable of causing haemorrhagic necrosis of animal tumours (Carswell *et al.*, 1975). It is also named cachectin because this molecule was originally isolated during a series of studies aimed at addressing the problem of cachexia in chronic disease states. Two proteins have been characterized and are available as recombinant DNA derived proteins. They are referred to as TNF (or TNF-alpha), which is predominantly derived from macrophages, and lymphotoxin (or TNF-beta) which is a product of activated lymphocytes.

In the late nineteenth century Coley (1893) observed that patients with streptococcal infection could have partial remission of concurrent malignancies. Although other European investigators noted a link between bacterial infections and cancer remission before Coley, this is the first observation related to the description of TNF. It has been subsequently demonstrated that the bacterial lipopolysaccaride (LPS) was able to trigger the production of a serum factor leading to tumour necrosis without causing shock and widespread tissue injury (Shear, 1944). Further investigation showed that LPS elicits the production of a host protein capable of inducing the haemorrhagic necrosis of tumours (O'Malley *et al.*, 1962). The molecule was characterised in the mid 1970s (Carswell *et al.*, 1975) and the material has been eventually sequenced and cloned in the mid 1980s (Pennica *et al.*, 1984).

TNF and lymphotoxin represent two important mediators in immunity and inflammation. They have a wide range of effects, including modulation of properties of vascular endothelium, induction of other cytokines, induction of antiviral activity, stimulation of bone resorption, angiogenesis and fibroblast growth. Following a brief description of the general properties of TNF, I will analyse the basic functions of this cytokine, notably: (1) its role in tumour cell cytotoxicity and/or growth; (2) its immuno-modulatory activity; and (3) its role in inflammatory responses.

Received 26 June 1989; and in revised form 10 October 1989.

### General properties of TNF

The production of TNF is not unique to a particular cell type. In fact, although the major source of this cytokine is macrophages (Beutler & Cerami, 1987; Le & Vilcek, 1987; Aggarwel *et al.*, 1985), the molecule can also be released by other cell types. These include monocytes stimulated by gamma interferon (Beutler & Cerami, 1987), T lymphocytes and T cell lines following stimulation with phorbol esters and anti-CD3 antibodies in different combinations (Granger & Williams, 1968; Cutri *et al.*, 1987; Scheurich *et al.*, 1987; Sung *et al.*, 1988*a*; Turner *et al.*, 1987), B lymphocytes (Williamson *et al.*, 1983; Sung *et al.*, 1988*b*), large granular lymphocytes (Ostensen *et al.*, 1987; Peters *et al.*, 1986) and mast cells (Young *et al.*, 1987).

The challenge with LPS appears to be the classical inducing agent for the release of TNF (Nedwin *et al.*, 1985*b*). Other stimulators acting *in vivo* include BCG, *Corynebacterium parvum*, *Brucella abortus* and interferon gamma (Nedwin *et al.*, 1985*b*; Old, 1985; Clark, 1982). As far as the regulation of the production of TNF *in vitro* is concerned, many stimuli cause cells to release TNF, including the binding of immunecomplexes and phagocytosis by mononuclear phagocytes, interferon gamma, interleukin-2 (IL-2), CSF-1, endotoxin, phorbol esters and viruses (Warren & Ralph, 1986; Strieter *et al.*, 1989*b*; Beutler & Cerami, 1987, 1988). Prostaglandins have demonstrated a suppressive effect on the release of macrophage-derived TNF production (Kunkel *et al.*, 1986, 1988) and glucocorticoids have been proved to suppress the transcription of TNF (Remick *et al.*, 1989). Peripheral blood monocytes and macrophages from different tissues exhibit both a different ability to express and release TNF after *in vitro* challenge and a different responsiveness to immunomodulators (Martinet *et al.*, 1989; Strieter *et al.*, 1989*a*). A differential regulation of TNF-alpha in human alveolar macrophages and peripheral blood monocytes has been documented (Strieter *et al.*, 1989*b*). In fact, prostaglandins and corticosteroids serve as potent regulators of LPS-induced TNF from peripheral blood monocytes, while alveolar macrophages are relatively refractory to these suppressive immunomodulating agents (Strieter *et al.*, 1989*b*).

The genes for both TNF-alpha and TNF-beta are separated by about 1 kb of DNA on chromosome 6 within the major histocompatibility complex (Nedwin *et al.*, 1985*a*; Spies *et al.*, 1986). The expression of TNF mRNA encodes a precursor of 233 amino acids that is processed to a mature non-glycosylated protein of 17,300 Da containing a single disulphide linkage (Wang *et al.*, 1985). Recent evidence has been provided favouring the suggestion that TNF-alpha may exist in dimeric or trimeric forms, each subunit of which consisting of an anti-parallel beta-sandwich (Smith & Baglioni, 1987; Jones *et al.*, 1989). The main chain fold of a TNF subunit shows a remarkable similarity to the 'jelly-roll' structural motif characteristics of viral coat proteins (Jones *et al.*, 1989).

Different cell types, usually following *in vitro* activation, possess the receptors for TNF, including macrophages, lymphocytes, polymorphonuclear cells, fibroblasts, endothelial cells, synovial cells, muscle cells, adipocytes, myeloblasts and tumour cells (Beutler *et al.*, 1985*b*; Beutler & Cerami, 1988;

Kull *et al.*, 1985). However, there is no correlation between the number of receptors and cell susceptibility, the cytolytic effect of TNF being dependent on the affinity of specific receptors (Kull *et al.*, 1985; Tsujimoto *et al.*, 1986; Lehemann *et al.*, 1986). For instance, polymorphonuclear leukocytes possess as much TNF receptors as tumour cell lines susceptible to cytolysis but they are not lysed (Larrick *et al.*, 1987; Tsujimoto *et al.*, 1986; Ruggiero *et al.*, 1987). Internalisation of TNF and/or its receptors appears to be required for mediation of cytotoxicity, with the ligand being degraded via a lysosomally dependent mechanism (Baglioni *et al.*, 1985; Kull *et al.*, 1985). Despite the fact that TNF and IL-1 are cytokines with multiple overlapping activities, TNF does not compete with the IL-1 binding to its receptors (Matsushima *et al.*, 1986).

### The activity on tumour cells

From an historical point of view, TNF was first identified for its anti-cancer activity. The biological activity of TNF was detected both by its *in vitro* cytotoxic effects on certain sensitive target cells, e.g. L-929 fibroblast-like line and U937 cells, and by its ability to induce the necrosis of Meth A sarcoma subcutaneously transplanted in mice. The story of TNF begins in the late nineteenth century when Coley had some success treating cancer patients by infecting them with live bacteria. It must be remembered, however, that at that time, i.e. the pre-antibiotics era, it was difficult to control infections resulting from bacteria. To prevent this inconvenience, the above investigator developed the so-called Coley's toxins, i.e. filtered supernatants from cultures of erisypeles lesions and *Bacillus prodigius*. As a matter of fact, these toxins could be administered without evidence of infections and the overall results were impressive (Coley Nauts *et al.*, 1953). Radiotherapy and chemotherapy then became available and, as a consequence, this type of approach was completely abandoned.

Availability of highly purified TNF prepared by recombinant DNA techniques allowed a better clarification of biological effects of TNF on tumour cell lines. A wide variability of behaviour has been displayed by different tumour cell lines with regard to the cytotoxic or cytostatic action of this molecule (Ortaldo *et al.*, 1986; Sugarman *et al.*, 1985; Wang *et al.*, 1985; Tsujimoto *et al.*, 1985). This variability has been proved to be independent of the number or affinity of TNF receptors, suggesting that a defect in the signal transduction mechanisms leading to the cytotoxic response might take place in unsensitive cells (Shepard & Lewis, 1988). Free radical generation during the TNF dependent conversion of arachidonic acid to prostaglandins and intracellular release of lysosomal enzymes have been thought to represent the crucial mechanisms accounting for the efficacy of killing capacity (Ruddle, 1987). The resistance of normal cells and many of the tumour cell lines to TNF is not due to a lack of TNF receptors or to a low binding affinity for the ligand, but to the absence of some biochemical signals elicited in sensitive cells after TNF binding to the receptors that are responsible for the cytolytic action (Sugarman *et al.*, 1985). Interestingly, the TNF resistance might be associated with the production of TNF by the resistant cells (Rubin *et al.*, 1986; Spriggs *et al.*, 1987). It has also been suggested that the transforming growth factors which are produced by different tumour cell lines may protect the tumour cell from destruction by TNF-alpha *in vivo* (Shepard & Lewis, 1988).

The finding that TNF can also destroy tumours *in vivo* even in the absence of a direct lytic effect of neoplastic cells *in vitro* has led to the hypothesis that TNF is able to display an indirect action. In fact, experimentally induced neoplasias generated from tumour cells not susceptible to the action of TNF *in vitro* are rapidly destroyed. Since tumour destruction takes place only in vascularised neoplasias, the indirect action seems to be mediated by the efficacy of TNF on the vascular endothelium of the tumour circulation (Palladino *et al.*, 1987.)

TNF has demonstrated a selective toxicity for leukaemic cells in myeloid leukaemias (Prince *et al.*, 1987). In this regard, the colony formation by clonogenic cells freshly derived from patients with acute myelogenous leukaemia, myeloid blast crisis of chronic myelogenous leukaemia, or chronic myelomonocytic leukaemia, was suppressed to various degrees by the presence of TNF-alpha. This suggests that the action of rTNF-alpha in myelogenous leukaemias could be exploited therapeutically and the dose–time response relationship should be considered in designing treatment schedules (Beran *et al.*, 1988).

Interestingly, TNF has also been demonstrated to act as a tumour growth factor in a dose-dependent manner for chronic B-cell malignancies and in particular for leukaemic hairy cells (Cordingley *et al.*, 1988*a*; Buck *et al.*, 1988; Trentin *et al.*, 1989). It has been shown to promote the proliferation of leukaemic cells and to induce TNF mRNA protein, thus supporting the concept of an autocrine model of tumour cell proliferation (Cordingley *et al.*, 1988*a*).

TNF has also been shown to modify the susceptibility of leukaemic cells to the lysis by autologous or allogenic cytotoxic lymphocytes. In fact, the demonstration has been provided that TNF, in association with INF-γ, increases the susceptibility of hairy cell leukaemia to natural killer (NK) cell lysis. This synergism is not mediated by a INF-γ induced increase in TNF receptors on hairy cells and therefore it seems to occur at a post-receptor level (Cordingley *et al.*, 1988*b*).

### The inflammatory activity

Several cell-to-cell communications are crucial during the initiation, maintenance and resolution of specific foci of inflammation. Lymphokines act as local mediators of cellular homeostasis and TNF plays a key role among these various cytokines.

One of the most important events that occurs during a local immune inflammatory response is represented by the effect of TNF on the endothelial surface. It is well known that the endothelial cell does not represent a bystander target cell but plays a crucial role during the immune responses. In this regard, TNF has been shown to stimulate the angiogenesis (Leibovich *et al.*, 1987) and to alter the endothelial cell responsiveness (Gamble *et al.*, 1985; Broudy *et al.*, 1987). In addition, TNF stimulates human vascular endothelial cells to release neutrophil chemotactic factors and to promote the transendothelial neutrophil influx (Moser *et al.*, 1988; Strieter *et al.*, 1988). It is worth mentioning that other cytokines, particularly IL-1 and INF-α, β, and γ, play an important role in regulating the endothelial responsiveness (Pober *et al.*, 1986; Moser *et al.*, 1988). Furthermore, TNF increases the expression of class I major histocompatibility complex (MHC) antigens on the vascular endothelium surface (Gamble *et al.*, 1985; Pohlman *et al.*, 1986). It also increases the production of procoagulants and down-regulates the production of thrombomodulin, thus converting vascular endothelium to a procoagulant surface (Stern & Nawaroth, 1986; Bevilacqua *et al.*, 1986). In fact, thrombomodulin binds to serum proteins S and C to promote local anti-coagulation. This finding, in association with the capacity of TNF to induce inflammatory cell adherence to vessel walls (Nawroth & Stern, 1986; Taylor *et al.*, 1987), represents an additional factor contributing to a coagulant state with cessation of blood flow and then leading to tissue necrosis.

TNF induces the trapping of neutrophils in localised areas, thus initiating the inflammatory response. In fact, TNF has chemotactic activity that may serve to recruit phagocytic cells from the blood compartment to amplify resistance against noxious agents (Ming *et al.*, 1987). During an inflammatory reaction, TNF allows neutrophils to respond more efficiently to an invasive agent by increasing their property to phagocytose (Klebanoff *et al.*, 1986) and by activating the superoxide anion production (Larrick *et al.*, 1987). Once the immune process has been initiated, TNF induces an oxidative burst, degranulation and increased phagocytic activity (Shalaby *et al.*, 1985; Klebanoff *et al.*, 1986).

TNF induces fever initially by increasing prostaglandin $E_2$ synthesis in the hypothalamus and subsequently by triggering the production of IL-1 (Dinarello *et al.*, 1986; Nawroth *et al.*, 1987). Other factors produced in response to the TNF include platelet-derived growth factor (Hajjar *et al.*, 1987), platelet-activating factor (Camussi *et al.*, 1987), prostacyclin (Kawakami *et al.*, 1986), osteoclasts activating factor (Dewhirst *et al.*, 1985; Bertolini *et al.*, 1986), and haematopoietic growth factors, including G-CSF and GM-CSF (Broudy *et al.*, 1987; Munker *et al.*, 1986; Trinchieri *et al.*, 1986; Zucali *et al.*, 1988).

TNF has also been implicated in the remodelling of connective tissue through an action on fibroblasts. In fact, TNF induces fibroblasts to grow (Vilcek *et al.*, 1986) and to produce IL-1 (Le *et al.*, 1987), colony stimulating factors (Zucali *et al.*, 1987), interferon beta-2 (Van Damme *et al.*, 1987) and glycosaminoglycans (Elias *et al.*, 1988). TNF has been found to be capable of stimulating collagenase and $PGE_2$ production by isolated synovial cells and dermal fibroblasts (Dayer *et al.*, 1985).

The evidence that mice passively immunised against the hormone are protected against the lethal effect of lypopolysaccaride suggested a primary role of TNF as a mediator of endotoxic shock (Beutler *et al.*, 1985*c*). When TNF became available in the recombinant form, and thus in preparations free of contaminating endotoxin, it was possible to demonstrate that TNF itself is able to induce the shock and the tissue injury usually associated with endotoxaemia (Tracey *et al.*, 1985).

Rats infused with recombinant TNF presented with diarrhoea, piloerection, haemoconcentration, shock, metabolic acidosis and hyperglycaemia followed by hypoglycaemia. Leucostasis, oedema, ischaemic and haemorrhagic lesions have been documented in different organs, including the lung, kidney, adrenal tissue, pancreas and gastrointestinal tract. Furthermore, it has been subsequently demonstrated that non-human primates can be protected against the lethal effects of endotoxin injection by pre-treatment with anti-TNF antibody (Tracey *et al.*, 1987). TNF has also been demonstrated to contribute to the pathogenesis of septic acute lung injury by producing increased pulmonary permeability and oedema (Stephens *et al.*, 1988).

The observation that trypanosome-infected rabbits develop an hypertriglyceridaemia associated with suppression of the enzyme lipoprotein lipase (Rouzer *et al.*, 1980) led to the discovery that a murine macrophage mediator, released under the action of LPS or other invasive stimuli, was able to suppress the lipoprotein lipase expression in the fatty tissues of mice and in cultured adipocytes (Kawakami & Cerami, 1981; Kawakami *et al.*, 1987). This factor was called cachectin and subsequent evaluations revealed that the sequence of cachectin and TNF were indistinguishable (Beutler *et al.*, 1985*a*). As a matter of fact, in experimental models it has been demonstrated that nude mice injected with cells constitutionally secreting TNF develop anorexia, weight loss and anaemia (Oliff *et al.*, 1987).

### The immunomodulatory activity

TNF has been demonstrated to display a series of species-specific effects on different cell types and functions. With regard to the monocyte/macrophage lineage, TNF provides important mechanisms to augment the effector activities of these cells at inflammatory foci. In particular, TNF enhances the cytotoxicity of macrophages (Philip *et al.*, 1986), induces the synthesis of interleukin-1 (Bachwich *et al.*, 1986*a*) and the expression of Fc receptors (Hoffman & Weinberg, 1987), and of Ia antigens (Chang & Lee, 1986). TNF also enhances the production of hydrogen peroxide (Hoffman & Weinberg, 1987), the synthesis of increased levels of platelet activating factor and prostaglandin $E_2$ (Bachwich *et al.*, 1986*a*; Camussi *et al.*, 1987).

The action of TNF on lymphocytes is displayed after the initial stimulation of T cells because resting T lymphocytes appear to lack the TNF receptors (Kull *et al.*, 1985). TNF can stimulate T lymphocytes in a dose-dependent manner (Zucali *et al.*, 1987) and it also modulates the proliferation and differentiation of B lymphocytes (Jelinek & Lipskey, 1987; Kashiwa *et al.*, 1987; Kehrl *et al.*, 1987). TNF enhances the IL-2R expression on a lymphoblastic null-cell leukaemic line in a fashion similar to that of IL-1 (Lee *et al.*, 1987). High concentrations of TNF induce T lymphocytes to release interferon gamma and TNF provides a synergistic effect with IL-2 in the generation of LAK cells (Owen-Schaub *et al.*, 1988; Chouaib *et al.*, 1988; Yang *et al.*, 1989).

TNF induces neutrophils to produce hydrogen peroxide (Shau, 1988) and stimulates human vascular endothelial cells to promote transendothelial neutrophil passage (Moser *et al.*, 1988).

TNF has been demonstrated to exert an anti-viral effect *in vitro*, which is mediated through the induction of IFN-beta. In fact, Kohase *et al.* (1986) and Mestan *et al.* (1986) showed that recombinant human TNF can produce an anti-viral effect in human diploid fibroblasts. This action of TNF could be abolished in the presence of antiserum specific for IFN-beta. By contrast, Wong & Goeddel (1986) suggested that the anti-viral action of TNF does not involve IFN as an intermediate. It has been demonstrated that cachectin/TNF selectively kills cells infected with herpes simplex virus (Koff & Fann, 1986), thus attributing a protective role to TNF. TNF-alpha is also able to stimulate the HIV enhancer by activation of the NF-kB transcription factor (Osborn *et al.*, 1989).

### Diseases in which an increase of TNF has been found

As far as experimental models are concerned, TNF has been proved to play an important role in the pathogenesis of cerebral malaria (Grau *et al.*, 1987) and to represent an effector of the skin and gut lesions of the acute phase of graft versus host disease (Piguet *et al.*, 1987). Furthermore, a relationship between BCG-induced granulomas and TNF synthesis has been recently reported (Kindler *et al.*, 1989). In these murine models, the *in vivo* treatment with an anti-TNF antibody resulted in an almost complete prevention of the above quoted lesions (Grau *et al.*, 1987; Piguet *et al.*, 1987; Kindler *et al.*, 1989). These latter findings suggest the hypothesis that the therapeutic possibilities of antibodies or specific antagonists against TNF should be taken into account.

The pleiomorphic effects of TNF on different target cells place this molecule in a pivotal role in modulating acute and chronic disease states. For this reason, the levels of TNF activity have been evaluated in different clinical conditions. No TNF activity was detected in blood cell extracts (Hofsli *et al.*, 1988) but TNF mRNA may be present *in vivo* (Tovey *et al.*, 1988).

The role of TNF in the pathogenesis of the cachexia associated with human chronic diseases remains to be determined since TNF cannot be detected in the plasma of cachectic patients. This may be consequent to the low sensitivity of assays presently available (Beutler & Cerami, 1987). As a matter of fact, a discrepancy has been observed in different biological and enzymatic assays which still need to be solved (Balkwill *et al.*, 1987*a*; Duncombe *et al* 1988; Fomsgaard *et al.*, 1988; Munck Petersen & Moller, 1988).

Increased levels of TNF were associated with poor prognosis in patients with meningococcal infections (Waage *et al.*, 1987; Girardin *et al.*, 1988). Serum levels of TNF-alpha were positively correlated with the number of risk factors and negatively correlated with blood fibrinogen levels, thus indicating that TNF correlates with the severity of meningococcaemia in children (Girardin *et al.*, 1988). Lipopolysaccharide exposed monocytes from patients with previous Yersinia arthritis secrete significantly more TNF than controls (Repo *et al.*, 1988).

Increased plasma levels of TNF has been observed in patients with sarcoidosis (Bachwich *et al.*, 1986*b*; Spatafora *et al.*, 1989), parasitic infections (Scuderi *et al.*, 1986) and autoimmune disorders (Turner *et al.*, 87). Among patients with malignancies, high serum levels of TNF have been found in patients with cancer (Balkwill *et al.*, 1987), chronic lymphocytic leukaemia (Hahn *et al.*, 1989), and hairy cell leukaemia (Buck *et al.*, 1988; Lindemann *et al.*, 1989). In addition, TNF mRNA has been demonstrated on hairy cells stimulated *in vitro* with TNF (Cordingley *et al.*, 1988*a*). Furthermore, the potential role of TNF as the mediator responsible for the extensive marrow necrosis found in patients with cancer has been suggested (Knupp *et al.*, 1988).

Increased levels of TNF have been described in the serum of patients with AIDS; this finding has been claimed to be relevant to the pathogenesis of cachexia in this disease (Lahdevirta *et al.*, 1988). Moreover, TNF-alpha levels were significantly higher in supernatants obtained by monocytes isolated from asymptomatic HIV-infected patients as compared to normal controls (Wright *et al.*, 1988; Roux-Lombard *et al.*, 1989). In this regard, conflicting results have been reported in a previous paper by Ammann *et al.* (1987). An increase in TNF production by alveolar macrophages recovered from the bronchoalveolar lavage in HIV infected patients has also been demonstrated (Agostini *et al.*, 1989).

### *In vivo* use of TNF: present status and future directions

In rabbits it has been shown that TNF is cleared from the plasma with a half-life of 6–7 min (Beutler *et al.*, 1985*b*). Studies on the tissue distribution of labelled TNF after injection have demonstrated that liver, kidneys, skin and gastrointestinal tract take up most of the lymphokine (Beutler *et al.*, 1985*b*). Studies with TNF, in association with IFN-$\gamma$, have also been performed in experimental ovarian cancer showing a significant activity (Balkwill *et al.*, 1987). A regression of a murine sarcoma has been observed after *in vivo* treatment with recombinant human TNF and this effect has been found to be obtained via Lyt-2$^+$ cells (Asher *et al.*, 1989).

The evidence accumulated in the animal models that the immune system can be manipulated to mediate the regression of established growing tumours, coupled to the availability of TNF produced through recombinant DNA technology, has enabled the exploration of the potential therapeutical benefits of TNF as an anti-neoplastic agent in human beings.

Phase I studies, in which TNF-alpha was given in a variety of schedules (single dose, continuous 5 days infusion, daily for 5 days, etc.) with doses increasing from 1 to 400 $\mu g\, m^{-2}$, indicated that the maximum tolerated dose of TNF seems to be 200 $\mu g\, m^{-2}$ (Blick *et al.*, 1987; Chapman *et al.*, 1987; Creaven *et al.*, 1987, 1989; Sherman *et al.*, 1988). TNF was administered by the intravenous and subcutaneous rute (Chapman *et al.*, 1987). The half-line of rTNF administered intravenously was 20 min. Side-effects include fever, chills, rigor, fatigue, diarrhoea, headache, nausea and vomiting, severe hypotension, and fluid retention most likely consequent to a capillary-leakage syndrome similar to that described for IL-2 (Chapman *et al.*, 1987; Creaven *et al.*, 1987; Kimura *et al.*, 1987; Mortiz *et al.*, 1989; Sherman *et al.*, 1988). However, fluid accumulation is much less prominent with TNF as compared to the retention observed during IL-2 therapy. In a few patients a transient elevation of transaminases has been observed but hepatic toxicity did not appear to play a role in TNF dose limitation (Creaven *et al.*, 1987; Kimura *et al.*, 1987). Transient thrombocytopenia and leukopenia have also been observed (Sherman *et al.*, 1988). Minor changes were seen in haemostatic parameters (Chapman *et al.*, 1987). Side-effects clear rapidly after discontinuing TNF administration while the febrile reaction is reduced by pre-treatment with paracetamol or indomethacin (Moritz *et al.*, 1989). Caution has been recommended in treating patients with pre-existing hepatic function abnormalities, hypertension, hypotension or significant obstructive airway disease (Creaven *et al.*, 1989). In addition, the precise role of TNF in stimulating the growth of both normal and leukaemic B cells (Jelinek & Lipskey, 1987; Cordingley *et al.*, 1988*a*) needs to be further elucidated and taken into account.

As far as immunological functional parameters of blood cells from patients receiving recombinant human TNF are concerned, phase I studies demonstrated that TNF acts *in vivo* directly or indirectly on NK cells and monocytes by either inactivating their functional capacity or by absorbing the relevant cells to the endothelial cell layer, thus removing them from circulation (Kist *et al.*, 1988).

Once the time of injection and doses were been standardised, clinical trials were designed. Large studies are currently underway but, for the time being, no consensus has emerged from preliminary results concerning the clinical efficacy of this lymphokine in the treatment of malignancy, little evidence of TNF anti-tumour activity having been observed *in vivo*. Although partial remissions were documented in individual patients with colon and pancreatic cancer and B cell lymphomas, only a few clinically significant benefits have been observed (Blick *et al.*, 1987; Creaven *et al.*, 1989; Herrmann, 1989; Moritz *et al.*, 1989; Selby *et al.*, 1987; Sherman *et al.*, 1988) and the role of TNF as a single agent is not presently recommended. Perhaps the actual meaning of the experiences reported to date is not that we are ready for a widespread application of TNF to the therapy of cancer patients but that eventually we can successfully manipulate the cellular immune system in the defense against tumours. However, we are at the very beginning of this new era of treatment with biological response modifiers, in particular using TNF. In fact, while clinical experiences with other molecules (e.g. INFs and IL-2) are quite well documented and established, immunotherapy with TNF is still in its infancy and needs to be standardised, for the time being its use remaining experimental. A series of studies is now being undertaken to increase the therapeutic efficacy of TNF treatment.

One of the most promising approaches is represented by the use of TNF in association with other interleukins, and in particular with IL-2 since a synergism occurs between TNF-alpha and IL-2 in the generation of lymphokine activated killer (LAK) cells (Chouaib *et al.*, 1988; Owen-Schaub *et al.*, 1988; Matossian-Rogers *et al.*, 1989; Yang *et al.*, 1989). The interaction between IL-2 and TNF on LAK precursors results in a reduction of the IL-2 concentration required for the differentiation of granular lymphocytes into LAK cells. In fact, the addition of TNF-alpha to peripheral blood lymphocytes stimulated with suboptimal IL-2 concentrations can augment the cytotoxicity to levels observed with 10 times more IL-2 alone and this of course can limit adverse reactions. Furthermore, TNF-alpha either alone or in combination with IL-2 has been demonstrated to increase the generation of specific cytotoxic T lymphocytes (Nakano *et al.*, 1989; Whiteside *et al.*, 1989). Since the function, but not the toxicity, of these two molecules is synergistic, this piece of information may be clinically adapted to improve the safety and to achieve therapeutic efficacy of immunotherapy trials without appreciable toxicity. In view of these possibilities, it is worth mentioning that once LAK cells are stimulated by tumour targets they become able to secrete TNF (Chong *et al.*, 1989). The range of agents that act synergistically with TNF is not limited to biologicals; chemotherapic drugs may show similar synergism thus offering choices for clinical testing. However, no clinical data are available to substantiate these pre-clinical studies.

Other approaches to be considered to identify a regimen of TNF administration that will induce the desired immunological effects with acceptable levels of toxicity include the increase of maximal tolerated dose and prolonged infusion times which allow the application of higher doses. In this regard, the cell cycle dependence of TNF cytotoxicity *in vitro* (Darzynkiewicz *et al.*, 1984) indicates that different schedules of administration need to be explored accurately to determine whether continuous or interrupted availability of TNF is more effective. In addition, the possibility to maximise the accumulation of TNF at the site of tumour growth must be further explored since some remissions have been reported with trials of recombinant TNF after direct injection into the tumour (Taguchi, 1987).

For the time being, the intercellular network of mechanisms regulating the cytokines circuits *in vivo* is not sufficiently understood to allow us to predict the anti-tumour effect as well as adverse reactions induced by these immunotherapeutic approaches. The use of lymphokines as pharmacological agents is probably more complicated than initially thought (Paul, 1989) and further knowledge of the physiology of the immune system will undoubtedly shed light on this issue, thus enabling investigators to validate these new therapeutic strategies.

Supported by a grant from AIRC (Milan). The author wishes to thank Miss Alison Cessario for her help in the preparation of the manuscript.

### References

AGGARWAL, B.B., KOHR, W.J., HASS, P.E. & 7 others (1985). Human tumor necrosis factor: production, purification, and characterization. *J. Biol. Chem.*, **260**, 2345.

AGOSTINI, C., TRENTIN, L., POLETTI, V. & 5 others (1989). Alveolar macrophages from patients with HIV infection spontaneously release tumor necrosis factor. V International Conference on AIDS, Montreal, 4–9 June, p. 429.

AMMANN, A.J., PALLADINO, M.A., VOLBERING, P., ABRAMS, D., MARTIN, N.L. & CONANT, M. (1987). Tumor necrosis factor alpha and beta in acquired immunodeficiency syndrome (AIDS) and AIDS related complex. *J. Clin. Immunol.*, **7**, 481.

ASHER, A.L., MULE, J.J. & ROSENBERG, S.A. (1989). Recombinant human tumor necrosis factor mediates regression of a murine sarcoma in vivo via Lyt-2$^+$ cells. *Cancer Immunol. Immunother.*, **28**, 153.

BACHWICH, P.R., CHENSUE, S.W., LARRICK, J.W. & KUNKEL, S.L. (1986*a*). Tumor necrosis factor stimulates interleukin-1 and prostaglandin $E_2$ production in resting macrophages. *Biochem. Biophys. Res. Commun.*, **136**, 94.

BACHWICH, P.R., LYNCH, J.P. III, LARRICK, J.W., SPENGLER, M. & KUNKEL, S.L. (1986*b*). Tumor necrosis factor production by human sarcoid alveolar macrophages. *Am. J. Pathol.*, **125**, 421.

BAGLIONI, C., MCCANDLESS, S., TAVERMIER, J. & FIERS, W. (1985). Binding of human tumor necrosis factor to high affinity receptors on HeLa and lymphoblastoid cells sensitive to growth inhibition. *J. Biol. Chem.*, **260**, 13395.

BALKWILL, F.R., BURKE, F., TALBOT, D. & 5 others (1987*a*). Evidence for tumour necrosis factor/cachectin production in cancer. *Lancet*, **ii**, 1229.

BALKWILL, F.R., WARD, B.G., MOODIE, E. & FIERS, W. (1987*b*). Therapeutic potential of tumor necrosis factor-alpha and gamma-interferon in experimental human ovarian cancer. *Cancer Res.*, **47**, 4755.

BERAN, M., MCCREDIE, K.B., KEATING, M.J. & GUTTERMAN, J.U. (1988). Antileukemic effect of recombinant tumor necrosis factor an in vitro and its modulation by α and γ interferons. *Blood*, **72**, 728.

BERTOLINI, D.R., NEDWIN, G.E., BRINGMAN, T.S., SMITH, D.D. & MUNDY, G.R. (1986). Stimulation of bone resorption and inhibition of bone formation *in vitro* by human tumor necrosis factors. *Nature*, **319**, 516.

BEUTLER, B. & CERAMI, A. (1987). Cachectin: more than a tumor necrosis factor. *N. Engl. J. Med.*, **316**, 379.

BEUTLER, B. & CERAMI, A. (1988). Cachectin (tumor necrosis factor): a macrophage hormone governing cellular metabolism and inflammatory response. *Endocr. Rev.*, **9**, 57.

BEUTLER, B., GREENWALD, D., HULMES, J.D. & 5 others (1985*a*). Identity of tumor necrosis factor and the macrophage-secreted factor cachectin. *Nature*, **316**, 552.

BEUTLER, B., MILSARK, I.W. & CERAMI, A. (1985*b*). Cachectin/tumor necrosis factor: production, distribution, and metabolic fate in vitro. *J. Immunol.*, **135**, 3972.

BEUTLER, B., MILSARK, I.W. & CERAMI, A. (1985*c*). Passive immunization against cachectin/tumor necrosis factor protects mice from lethal effects of endotoxin. *Science*, **229**, 869.

BEVILACQUA, M.P., PROBER, J.S., MAJEAU, G.R., FIERS, W., COTRAN, R.S. & GIMBRNE, M.A. JR (1986). Recombinant tumor necrosis factor induces procoagulant activity in cultured human vascular endothelium: characterization and comparison with the actions of interleukin 1. *Proc. Natl Acad. Sci. USA*, **83**, 4533.

BLICK, M., SHERWIN, S.A., ROSEMBLUM, M. & GUTTERMAN, J. (1987). Phase I study of recombinant tumor necrosis factor in cancer patients. *Cancer Res.*, **47**, 2986.

BROUDY, V.C., HARLAN, J.M. & ADAMSON, J.W. (1987). Disparate effects of tumor necrosis factor-alpha/cachectin and tumor necrosis factor beta/lymphotoxin on hematopoietic growth factor production and neutrophil adhesion molecular expression by cultured human endothelial cells. *J. Immunol.*, **138**, 4298.

BUCK, C., DIGEL, W., SCHONIGER, W., STEFANIC, M., RAGHAVACHAR, A. & PROZSOLT, F. (1988). Tumour necrosis factor and hairy cell leukaemia. *Lancet*, **ii**, 402.

CAMUSSI, G., BUSSOLINO, F., SALVIDIO, G. & BAGLIONI, C. (1987). Tumor necrosis factor/cachectin stimulates peritoneal macrophages, polymorphonuclear leukocytes, and vascular endothelial cells to synthesize and release platelet activating factor. *J. Exp. Med.*, **166**, 1390.

CARSWELL, E.A., OLD, L.J., KASSEL, R.L., GREEN, S., FIORE, N. & WILLIAMSON, B.D. (1975). An edotoxin-induced serum factor that causes necrosis of tumors. *Proc. Natl Acad. Sci. USA*, **72**, 3666.

CHANG, R.J. & LEE, S.H. (1986). Effects of interferon-gamma and tumor necrosis factor-alpha on the expression of an Ia antigen on a murine macrophage line. *J. Immunol.*, **137**, 2853.

CHAPMAN, P.B., LESTER, T.J., CASPER, E.S. & 10 others (1987). Clinical pharmacology of recombinant human tumor necrosis factor in patients with advanced cancer. *J. Clin. Oncol.*, **5**, 1942.

CHONG, A.S.F., ALEKSIJEVIC, A., SCUDERI, P., HERSH, E.M. & GRIMES, W.J. (1989). Phenotypic and functional analysis of lymphokine-activated killer (LAK) cell clones. Ability of CD3 +, LAK cell clones to produce interferon-y and tumor necrosis factor upon stimulation with tumor targets. *Cancer Immunol. Immunother.*, **29**, 270.

CHOUAIB, S., BERTOGLIO, J., BLAY, J.Y., MARCHIOL-FOURNIGAULT, C. & FRADELIZI, D. (1988). Generation of lymphokine-activated killer cells: synergy between tumor necrosis factor and interleukin 2. *Proc. Natl Acad. Sci. USA*, **85**, 6875.

CLARK, I.A. (1982). Suggested importance of monokines in pathophysiology of endotoxic shock and malaria. *Klin. Wochenschr.*, **60**, 756.

COLEY, W.B. (1893). The treatment of malignant tumors by repeated inoculations of erysipelas: with a report of ten original cases. *Am. J. Med. Sci.*, **105**, 487.

COLEY NAUTS, H., FOWLER, G.A.A. & BOGATKO, F.H. (1953). A review of the influence of bacterial infection and of bacterial products (Coley's toxins) on malignant tumors in man. *Acta Med. Scand.*, **145**, suppl. 276, 29.

CORDINGLEY, F.T., BIANCHI, A., HOFFBRAND, A.V. & 6 others (1988*a*). Tumour necrosis factor as an autocrine tumour growth factor for chronic B-cell malignancies. *Lancet*, **i**, 969.

CORDINGLEY, F.T., HOFFBRAND, A.V. & BRENNER, M.K. (1988*b*). Cytokine-induced enhancement of the susceptibility of hairy cell leukemia lymphocytes to natural killer cell lysis. *Br. J. Haematol.*, **70**, 37.

CREAVEN, P.J., BRENNER, D.E., COWENS, J.W. & 6 others (1989). A phase I clinical trial of recombinant human tumor necrosis factor given daily for five days. *Cancer Chemother. Pharmacol.*, **23**, 186.

CREAVEN, P.J., PLAGER, J.E., DUPERE, S. & 4 others (1987). Phase I clinical trial of recombinant human tumor necrosis factor. *Cancer Chemother. Pharmacol.*, **20**, 137.

CUTRI, M.C., MURPHY, M., COSTA-GIOMI, M.P., WEINMANN, R., PERUSSIA, B. & TRINCHERI, G. (1987). Independent regulation of tumor necrosis factor and lymphotoxin production by human peripheral blood lymphocytes. *J. Exp. Med.*, **165**, 1581.

DARZYNKIEWICZ, Z., WILLIAMSON, B., CARSWELL, E.A. & OLD, L.J. (1984). Cell cycle-specific effects of tumor necrosis factor. *Cancer Res.*, **44**, 83.

DAYER, J.M., BEUTLER, B. & CERAMI, A. (1985). Cachectin/tumor necrosis factor stimulates collagenase and prostaglandin $E_2$ production by human synovial cells and dermal fibroblasts. *J. Exp. Med.*, **162**, 2163.

DEWHIRST, F.E., STASHENKO. P.P., MOLE, J.E. & TSURUMACHI, T. (1985). Purification and partial sequence of human osteoclast-activating factor: identity with interleukin-1$\beta$. *J. Immunol.*, **135**, 2562.

DINARELLO, C.A., CANNON, J.G., WOLFF, S.M. & 6 others (1986). Tumor necrosis factor (cachectin) is an endogenous pyrogen and induces production of interleukin-1. *J. Exp. Med.*, **163**, 1433.

DUNCOMBE, A.S., GOTTLIEB, D.J., BIANCHI, A. & BRENNER, M.K. (1988). Bioactivity and immunoreactivity of tumour necrosis factor in cancer patients. *Lancet*, **i**, 248.

ELIAS, J.A., KROL, R.C., FUENDLICH, B. & SAMPSON, P.M (1988). Regulation of human lung fibroblast glycosaminoglycan production by recombinant interferons, tumor necrosis, and lymphotoxin. *J. Clin. Invest.*, **81**, 325.

FOMSGAARD, A., WORSAAE, H. & BENDTZEN, K. (1988). Detection of tumor necrosis factor from lipopolysaccaride-stimulated human mononuclear cells by enzyme-linked immunosorbent assay and cytotoxicity bioassay. *Scand. J. Immunol.*, **27**, 143.

GAMBLE, J.R., HARLAN, J.M., KLEBANOFF, S.J. & VADAS, M.A. (1985). Stimulation of the adherence of neutrophils to umbilical vein endothelium by human recombinant tumor necrosis factor. *Proc. Natl Acad. Sci. USA*, **82**, 8667.

GIRARDIN, E., GRAU, G.E., DAYER, J.M., ROUX-LOMBARD, P., The J5 Study Group & LAMBERT, P.H. (1988). Tumor necrosis factor and interleukin-1 in the serum of children with severe infectious purpura. *N. Engl. J. Med.*, **319**, 397.

GRANGER, G.A. & WILLIAMS, T.W. (1968). Lymphocyte cytotoxicity *in vitro:* Activation and release of a cytotoxic factor. *Nature*, **218**, 1253.

GRAU, G.E., FAJARDO, L.F., PIGUET, P.F., ALLET, B., LAMBERT, P.H. & VASSALLI, P. (1987). Tumor necrosis factor (Cachectin) as an essential mediator in murine cerebral malaria. *Science*, **237**, 1210.

HAHN, T., KUSMINSKY, G., BASSOUS, L., BARAK, Y. & BERREBI, A. (1989). Tumour necrosis factor in B chronic lymphocytic leukemia. *Br. J. Haematol.*, **71**, 299.

HAJJAR, K.A., HAJJAR, D.P., SILVERSTEIN, R.L. & NACKMAN, R.L. (1987). Tumor necrosis factor-mediated release of platelet-derived growth factor from cultured endothelial cells. *J. Exp. Med.*, **166**, 235.

HERRMANN, F. (1989). Cytokines in cancer therapy. *J. Cancer Res. Clin. Oncol.*, **115**, 101.

HOFFMAN, M. & WEINBERG, J.B. (1987). Tumor necrosis factor-alpha induces hydrogen peroxide production and Fc receptor expression, but not increased Ia antigen expression by peritoneal macrophages. *J. Leuk. Biol.*, **42**, 704.

HOFSLI, E., LAMVIK, J. & NISSEN-MEYER, J. (1988). Evidence that tumor necrosis factor (TNF) is not constitutively present *in vivo*. The association of TNF with freshly isolated monocytes reflects a rapid *in vitro* production. *Scand. J. Immunol.*, **28**, 435.

JELINEK, D.F. & LIPSKEY, P.E. (1987). Enhancement of human B cell proliferation and differentiation by tumor necrosis factor-alpha and interleukin-1. *J. Immunol.*, **139**, 2970.

JONES, E.Y., STUART, D.I. & WALKER, N.P.C. (1989). Structure of tumour necrosis factor. *Nature*, **238**, 225.

KASHIWA, H., WRIGHT, S.C. & BONAVIDA, B. (1987). Regulation of B cell maturation and differentiation. I. Suppression of pokeweed-mitogen induced B cell differentiation by tumor necrosis factor. *J. Immunol.*, **138**, 1383.

KAWAKAMI, M. & CERAMI, A. (1981). Studies of endotoxin-induced decrease in lipoprotein lipase activity. *J. Exp. Med.*, **154**, 631.

KAWAKAMI, M., ISHIBASHI, S., OGAWA, H., MUROSE, T., TAKAKU, F. & SHIBATA, S. (1986). Cachectin/TNF as well as interleukin-1 induces prostacyclin synthesis in cultured vascular endothelial cells. *Biochem. Biophys. Res. Commun.*, **141**, 482.

KAWAKAMI, M., PEKALA, P.H., LANE, M.D. & CERAMI, A. (1987). Lipoprotein lipase suppression in 3T3-L1 cells by an entotoxin-induced mediator from exudate cells. *Proc. Natl Acad. Sci. USA*, **79**, 912.

KEHRL, J.H., MILLER, A. & FAUCI, A. (1987). Effect of tumor necrosis factor alpha on mitogen-activated human B cells. *J. Exp. Med.*, **166**, 786.

KIMURA, K., TAGUCHI, T., URUSHIZAKI, I., 14 others and the A-TNF Cooperative Study Group (1987). Phase I study of recombinant human tumor necrosis factor. *Cancer Chemother. Pharmacol.*, **20**, 223.

KINDLER, V., SAPPINO, A.P., GRAU, G.E., PIGUET, P.F. & VASSALLI, P. (1989). The inducing role of tumor necrosis factor in the development of bactericidal granulomas during BCG infection. *Cell*, **56**, 731.

KIST, A., HO, A.D., RATH, & 5 others (1988). Decrease of natural killer cell activity and monokine production in peripheral blood of patients treated with recombinant tumor necrosis factor. *Blood*, **72**, 344.

KLEBANOFF, S.J., VADAS, M.A., HARLAN, J.M. & 4 others (1986). Stimulation of neutrophils by tumor necrosis factor. *J. Immunol.*, **136**, 4220.

KNUPP, C., PEKALA, P.H. & CORNELIUS, P. (1988). Extensive bone marrow necrosis in patients with cancer and tumor necrosis factor activity in plasma. *Am. J. Hematol.*, **29**, 25.

KOFF, W.C. & FANN, A.V. (1986). Human tumor necrosis factor-alpha kills herpesvirus-infected but not normal cells. *Lymphokine Res.*, **5**, 215.

KOHASE, M., HENRIKSEN-DESTEFANO, D., MAY, L.T., VILCEK, J. & SEHGAL, P.B. (1986). Induction of $\beta_2$-interferon by tumor necrosis factor: a homeostatic mechanism in the control of cell proliferation. *Cell*, **45**, 659.

KUNKEL, S.L., SPENGLER, M., CHENSUE, S.W., LARRICK, J.W., KWON, G. & REMICK, D.G. (1988). Prostaglandin $E_2$ regulates macrophage-derived tumor necrosis factor gene expression. *J. Biol. Chem.*, **263**, 5380.

KUNKEL, S.L., WIGGINS, R.W., CHENSUE, S.W. & LARRICK, J.W. (1986). Regulation of macrophage tumor necrosis factor production by prostaglandin $E_2$. *Biochem. Biophys. Res. Commun.*, **137**, 404.

KULL, F.C., JACOBS, S. & CUATRECASAS, P. (1985). Cellular receptor for 125-I-labeled tumor necrosis factor: specific binding, affinity labeling, and relationship to sensitivity. *Proc. Natl Acad. Sci. USA*, **82**, 5756.

LAHDERIRTA, J., MAURY, C.P.J., TEPPO, A.M. & REPO, H. (1988). Elevated levels of circulating cachectin/tumor necrosis factor in patients with acquired immunodeficiency syndrome. *Am. J. Med.*, **85**, 289.

LARRICK, J.W., GRAHAM, D., TOY, K., LIN, L., SENYL, G. & FINDLEY, B.M., (1987). Recombinant tumor necrosis factor causes activation of human granulocytes. *Blood*, **69**, 640.

LE, J. & VILCEK, J. (1987). Tumor necrosis factor and interleukin-1: cytockines with multiple overlapping biological activities. *Lab. Invest.*, **56**, 234.

LE, J., WEINSTEIN, D., GUBLER, V. & VILCEK, J. (1987). Induction of membrane associated interleukin-1 by tumor necrosis factor in human fibroblasts. *J. Immunol.*, **134**, 895.

LEE, J.C., TRUNEH, A., SMITH, M.F. & TSANG, K.Y. (1987). Induction of interleukin 2 receptor (TAC) by tumor necrosis factor in YT cells. *J. Immunol.*, **139**, 1935.

LEHMANN, V. & DROGE, W. (1986). Demonstration of membrane receptors for human natural and recombinant $^{125}$I-labeled tumor necrosis factor on HeLa cell clones and their role in tumor cell sensitivity. *Eur. J. Biochem.*, **158**, 1.

LEIBOVICH, S.J., POLVERINI, P.J., SHEPARD, H.M., WISEMAN, D.M., SHIVELY, V. & NUSEIR, N. (1987). Macrophage-induced angiogenesis is mediated by tumor necrosis factor (TNF-$\alpha$). *Nature*, **329**, 630.

LINDEMANN, A., LUDWIG, W.D., OSTER, W., MERTELSMANN, R. & HERRMANN, F. (1989). High-level secretion of tumor necrosis factor-alpha contributes to hematopoietic failure in hairy cell leukemia. *Blood*, **73**, 880.

MARTINET, Y. YAMAUCHI, K. & CRYSTAL, R.G. (1988). Differential expression of the tumor necrosis factor/cachectin gene by blood and lung mononuclear phagocytes. *Am. Rev. Respir. Dis.*, **138**, 659.

MATOSSIAN-ROGERS, A., BROWNE, C., TURKISH, M., O'BYRNE, P. & FESTENSTEIN, H. (1989). Tumour necrosis factor-alpha enhances the cytolytic and cytostatic capacity of interleukin-2 activated killer cells. *Br. J. Cancer*, **59**, 573.

MATSUSHIMA, J., AKAHOSHI, T., YAMADA, M., FURUTANI, Y. & OPPENHEIM, J.J. (1986). Properties of a specific interleukin 1 (IL 1) receptor on human Epstein-Barr virus-transformed B lymphocytes: identity of the receptor for IL 1$\alpha$ and IL 1-$\beta$. *J. Immunol.*, **136**, 4496.

MESTAN, J., DIGEL, W., MITTNACHT, S. & 5 others (1986). Antiviral effects of recombinant tumor necrosis factor *in vivo*. *Nature*, **323**, 816.

MING, W.J.I., BERSANI, L. & MANTOVANI, A. (1987). Tumor necrosis factor is chemotactic for monocytes and polymorphonuclear leukocytes. *J. Immunol.*, **138**, 1469.

MORITZ, T., NIEDERLE, N., BAUMANN, J. & 6 others (1989). Phase I study of recombinant human tumor necrosis factor alpha in advanced malignant disease. *Cancer Immunol. Immunother.*, **28**, 144.

MOSER, R., SCHLEIFFENBAUM, B., GROSCURTH, P. & FEHR, J. (1988). Interleukin 1 and tumor necrosis factor stimulate human vascular endothelial cells to promote transendothelial neutrophil passage. *J. Clin. Invest.*, **83**, 444.

MUNCK PETERSEN, C. & MOLLER, B.K. (1988). Immunologic reactivity and bioactivity of tumour necrosis factor. *Lancet*, **i**, 934.

MUNKER, R., GASSON, J., OGAWA, M. & KOEFFLER, H.P. (1986). Recombinant human tumor necrosis factor induces production of granulocyte monocyte colony-stimulating factor. *Nature*, **323**, 816.

NAKANO, K., OKUGAWA, K., FURUICHI, H., MATSUI, Y. & SOHMURA, Y. (1989). Augmentation of the generation of cytotoxic T lymphocytes against syngeneic tumor cells by recombinant human tumor necrosis factor. *Cell Immunol.*, **120**, 154.

NAWROTH, P.P., BANK, I., HANDLEY, D., CASSIMERIS, J., CHESS, J. & STERN, D. (1987). Tumor necrosis factor/cachectin interacts with endotoxin cell receptors to induce release of interleukin-1. *J. Exp. Med.*, **163**, 1363.

NAWROTH, P.P. & STERN, D. (1986). Tumor necrosis factor/cachectin interacts with endotoxin cell receptors to induce release of interleukin-1. *J. Exp. Med.*, **163**, 740.

NEDWIN, G.E., NAYLOR, S.L., SAKAGUCHI, A.Y. & 5 others (1985*a*). Human lymphotoxin and tumor necrosis factor genes: structure homology and chromosomal localization. *Nucleic Acid Res.*, **13**, 6361.

NEDWIN, G.E., SVEDERSKY, L.P., BRINGMAN, T.S., PALLADINO, M.A. & GOEDDEL, D.V. (1985*b*). Effect of interleukin 2, interferon γ, and mitogens on the production of tumor necrosis factors α and β. *J. Immunol.*, **135**, 2492.

OLD, L.I. (1985). Tumor necrosis factor (TNF). *Science*, **230**, 630.

OLIFF, A., DEFEO-JONES, D., BOYER, M. & 5 others (1987). Tumor secreting human TNF/cachectin induce cachexia in mice. *Cell*, **50**, 555.

O'MALLEY, W.E., ACHINSTEIN, B. & SHEAR, M.J. (1962). Action of bacterial polysaccharide on tumors. II. Damage of sarcoma 37 by by serum of mice treated with Serratia marcescens polysaccharide, and induced tolerance. *J. Natl Cancer Inst.*, **29**, 1169.

ORTALDO, J.R., RANSON, J.R., SAYERS, T.J. & HERBERMAN, R.B. (1986). Analysis of cytostatic/cytotoxic lymphokines: relationship of natural killer cytotoxic factor to recombinant lymphotoxin, recombinant tumor necrosis factor, and leukoregulin. *J. Immunol.*, **137**, 2857.

OSBORN, L., KUNKEL, S. & NABEL, G.J. (1989). Tumor necrosis factor-α and interleukin-1 stimulate the HIV enhancer by activation of the NF-kB transcription factor. *Proc. Natl Acad. Sci. USA*, **86**, 2336.

OSTENSEN, M.E., THIELE, D.L. & LIPSKY, P.L. (1987). Tumor necrosis factor-alpha enhances cytolytic activity of human natural killer cells. *J. Immunol.*, **138**, 4185.

OWEN-SCHAUB, L.B., GUTTERMAN, J. & GRIMM, E.A. (1988). Synergy of tumor necrosis factor and interleukin 2 in the activation of human cytotoxic lymphocytes: effect of tumor necrosis factor α and interleukin 2 in the generation of human lymphokine-activated killer cell cytotoxicity. *Cancer Res.*, **48**, 788.

PALLADINO, M.A. JR, SHALABY, M.F., KRAMER, S.M. & 9 others (1987). Characterization of the antitumor activities of human tumor necrosis factor-α and the comparison with other cytokines: induction of tumor specific immunity. *J. Immunol.*, **138**, 4023.

PAUL, W.E. (1989). Pleiotropy and redundancy: T cell-derived lymphokines in the immune response. *Cell*, **57**, 521.

PENNICA, D., NEDWIN, G.E., HAYFLICK, J.S. & 9 others (1984). Human tumor necrosis factor: precursor structure, expression and homology to lymphotoxin. *Nature*, **312**, 724.

PETERS, P.M., ORTALDO, J.R., SHALABY, M.R. & 8 others (1986). Natural killer-densitive targets stimulate production of TNF-alpha but not TNF-beta (lymphotoxin) by highly purified human peripheral blood large granular lymphocytes. *J. Immunol.*, **137**, 2592

PIGUET, P.F., GRAU, G.E., ALLET, B. & VASSALLI, P. (1987). Tumor necrosis factor/cachectin is an effector of skin and gut lesions of the acute phase of graft-vs.-host disease. *J. Exp. Med.*, **166**, 1280.

PHILIP, R. & EPSTEIN, L. (1986). Tumor necrosis factor as immunomodulator and mediator of monocyte cytotoxicity induced by itself, gamma interferon, and interleukin-1. *Nature*, **323**, 86.

POBER, J.S., GIMBRONE, M.A. JR, LAPIERRE, L.A. & 4 others (1986). Overlapping patterns of activation of human endothelial cells by interleukin 1, tumor necrosis factor, and immune interferon. *J. Immunol.*, **137**, 1893.

POHLMAN, T.H., STANNES, K.A., BEATTY, P.G., OCHS, H.D. & HARLAN, J.M. (1986). An endothelial cell surface factor(s) induced in vitro by lipopolysaccharide, interleukin 1, and tumor necrosis factor-alpha increases neutrophil adherence by a CDw18-dependent mechanism. *J. Immunol.*, **136**, 4548.

PRICE, G., BRENNER, M.K., PRENTICE, H.G. & NEWLANDS, A.J. (1987). Cytotoxic effects of tumor necrosis factor and gamma interferon on myeloid leukaemia blast cells. *Br. J. Cancer*, **55**, 287.

REMICK, D.G., STRIETER, R.M., LYNCH, J.P. III, NGUYEN, D., RSKANDARI, M. & KUNKEL, S.L. (1989). *In vivo* dynamics of murine tumor necrosis factor-alpha gene expression: kinetics of dexamethasone-induced suppression. *Lab. Invest.*, **60**, 766.

REPO, H.,JAATTELA, M., LEIRISALO-REPO, M. & HURME, M. (1988). Production of tumor necrosis factor and interleukin 1 by monocytes of patients with previous Yersinia arthritis. *Clin. Exp. Immunol.*, **72**, 410.

ROUZER, C.A. & CERAMI, A. (1980). Hypertriglyceridemia associated with Trypanosoma brucei infection in rabbits: role of defective triglyceride removal. *Mol. Biochem. Parasitol.*, **2**, 31.

ROUX-LOMBARD, P., MODOUX, C., CRUCHAUD, A. & DAYER, J.M. (1989). Purified blood monocytes from HIV 1 infected patients produce high levels of TNF alpha and IL-1. *Clin. Immunol. Immunopathol.*, **50**, 374.

RUBIN, B.Y., ANDERSON, S.L., SULLIVAN, S.A., WILLIAMSON, B.D., CARSWELL, E. & OLD, L.J. (1986). Nonhematopietic cells selected for resistance to tumor necrosis factor produce tumor necrosis factor. *J. Exp. Med.*, **164**, 1350.

RUDDLE, N.H. (1987). Tumor necrosis factor and related cytotoxins. *Immunol. Today*, **8**, 129.

RUGGIERO, V., LATHAM, K. & BAGLIONI, C. (1987). Cytostatic and cytotoxic activity of tumor necrosis factor on human cancer cells. *J. Immunol.*, **138**, 2711.

SCHEURICH, P., THOMA, B., UCER, U. & PFIZENMAIER, K. (1987). Immunoregulatory activity of recombinant human tumor necrosis factor (TNF)-alpha: induction of TNF receptors on human T cells and TNF-α-mediated enhancement of T cell responses. *J. Immunol.*, **138**, 1786.

SCUDERI, P., LAM, K.S., RYAN, K.J. & 6 others (1986). Raised serum levels of tumour necrosis factor in parasitic infections. *Lancet*, **ii**, 1364.

SELBY, P., HOBBS, S., VINER, C. & 8 others (1987). Tumour necrosis factor in man, clinical and biological observations. *Br. J. Cancer*, **56**, 803.

SHALABY, M.R., AGGAZWAL, B.B., RINDERKNECHT, E., SVEDERSKY, L.P., FINKLE, B.S. & PALLADINO, M.A. Jr (1985). Activation of human polymorphonuclear neutrophil functions by interferon-γ and tumor necrosis factors. *J. Immunol.*, **135**, 2069.

SHAU, H. (1988). Characteristics and mechanism of neutrophil-mediated cytostasis induced by tumor necrosis factor. *J. Immunol.*, **141**, 234.

SHEAR, M.J. (1944). Chemical treatment of tumors. IX. Reactions of mice with primary subcutaneous tumors to injection of a hemorrhage-producing bacterial polysaccharide. *J. Natl Cancer Inst.*, **4**, 461.

SHEPARD, H.M. & LEWIS, G.D. (1988). Resistance of tumor cells to tumor necrosis factor. *J. Clin. Immunol.*, **8**, 333.

SHERMAN, M.L., SPRIGGS, D.R., ARTHUR, K.A., IMAMURA, K., FREI, E. III & KUFE, D.W. (1988). Recombinant human tumor necrosis factor administered as a five-day continuous infusion in cancer patients: phase I toxicity and effects on lipid metabolism. *J. Clin. Oncol.*, **6**, 344.

SMITH, R.A. & BAGLIONI, C. (1987). The active form of tumor necrosis factor is a trimer. *J. Biol. Chem.*, **261**, 6951.

SPATAFORA, M., MERENDINO, A., CHIAPPARA, G. & 4 others (1989). Lung compartmentalization of increased TNF releasing ability by mononuclear phagocytes in pulmonary sarcoidosis. *Chest*, **96**, 542.

SPIES, T., MORTON, C.C., NEDOSPASOV, S.A., FIERS, W., PIOUS, D. & STROMINGER, J.L. (1986). Genes for tumor necrosis factor α and β are linked to the human major histocompatibility complex. *Proc. Natl Acad. Sci. USA*, **83**, 8699.

SPRIGGS, D., IMAMURA, K., RODRIGUEZ, C., HORIGUCHI, J. & KUFE, D.W. (1987). Induction of tumor necrosis factor expression and resistance in human breast tumor cell line. *Proc. Natl Acad. Sci. USA*, **84**, 6563.

STEPHENS, K.E., ISHIZAKA, A., LARRICK, J.W. & RAFFIN, T.A. (1988). Tumor necrosis factor causes increased pulmonary permeability and edema. *Am. Rev. Respir. Dis.*, **137**, 1364.

STERN, D.M. & NAWAROTH, P.P. (1986). Modulation of endothelial cell hemostatic properties by tumor necrosis factor. *J. Exp. Med.*, **163**, 740.

STRIETER, R.M., KUNKEL, S.L., SHOWELL, H.J. & MARKS, R.M. (1988). Monokine-induced gene expression of a human endothelial cell-derived neutrophil chemotactic factor. *Biochem. Biophys. Res. Commun.*, **156,** 1340.

STRIETER, R.M., REMICK, D.G., LYNCH, J.P. III & 4 others (1989*a*). Differential regulation of tumor necrosis factor-alpha in human alveolar macrophages and peripheral blood monocytes: a cellular and molecular analysis. *Am. J. Resp. Cell Mol. Biol.*, **1,** 57.

STRIETER, R.M., REMICK, D.G., LYNCH, J.P. III, SPENGLER, R. & KUNKEL, S.L. (1989*b*). Interleukin-2 induced tumor necrosis factor-alpha (TNF-a) gene in human alveolar macrophages and blood monocytes. *Am. Rev. Respir. Dis.*, **139,** 335.

SUGARMAN, B.J., AGGARWAL, B.B., HASS, P.E., FIGARI, I.S., PALLADINO, M.A. & SHEPARD, H.M. (1985). Recombinant human tumor necrosis factor-alpha: effects on proliferation of normal and transformed cells *in vitro*. *Science*, **230,** 943.

SUNG, S.J., BJORNDAHL, J.M., WANG, C.Y., KAO, H.T. & FU, S.M. (1988*a*). Production of tumor necrosis factor/cachectin by human T cell lines and peripheral blood T lymphocytes stimulated by phorbol myristate acetate and anti-CD3 antibody. *J. Exp. Med.*, **167,** 937.

SUNG, S.J., JUNG, L.K.L., WALTERS, J.A., CHEN, W., WANG, C.Y. & FU, S.M. (1988*b*). Production of tumor necrosis factor/cachectin by human B cell lines and tonsillar B cells. *J. Exp. Med.*, **168,** 1539.

TAYLOR, F.B. Jr, CHANG, A., ESMON, C.T. & 4 others (1987). Protein C prevents the coagulopathic and lethal effects of Escherichia Coli infusion in the baboon. *J. Clin. Invest.*, **79,** 918.

TAGUCHI, T. (1987). Clinical studies on recombinant human tumor necrosis factor. *Immunobiology*, **175,** 37.

TOVEY, M.G., CONTENT, J., GRESSER, I. & 7 others (1988). Genes for IFN-beta-2 (IL-6), tumor necrosis factor, and IL-1 are expressed at high levels in the organs of normal individuals. *J. Immunol.*, **141,** 3106.

TRACEY, K.J., BEUTLER, B., LOWRY, S.F. & 9 others (1985). Shock and tissue injury induced by recombinant human cachectin. *Science*, **234,** 470.

TRACEY, K.J., FONG, Y., HESSE, D.G. & 5 others (1987). Anti-cachectin/TNF monoclonal antibodies prevent septic shock during lethal bacteraemia. *Nature*, **330,** 662.

TRENTIN, L., ZAMBELLO, R., PIZZOLO, G. & 5 others (1989). Tumor necrosis factor-α and B cell growth factor induce leukemic hairy cells to proliferate *in vitro*. *Cancer Prev. Det.* (in the press).

TRINCHIERI, G., KOBAYASHI, M., ROSEN, M., LOUDON, R., MURPHY, M. & PERUSSIA, B. (1986). Tumor necrosis factor and lymphotoxin induce differentiation of human myeloid cell lines in synergy with immune interferon. *J. Exp. Med.*, **164,** 1206.

TSUJIMOTO, M., FEINMAN, R., KOHASE, M. & VILCEK, J. (1986). Characterization and affinity crosslinking of receptors for tumour necrosis factor on human cells. *Arch. Biochem. Biophys.*, **249,** 563.

TSUJIMOTO, M., YIP, Y.K. & VILCEK, J. (1985). Tumor necrosis factor: specific binding and internalization in sensitive and resistant cells. *Proc. Natl Acad. Sci. USA*, **82,** 7626.

TURNER, M., LONDEI, M. & FELDMANN, M. (1987). Human T cells from auto-immune and normal individuals can produce tumor necrosis factor. *Eur. J. Immunol.*, **17,** 1807.

VAN DAMME, J., OPDENAKKER, G., SIMPSON, R.J. & 5 others (1987). Identification of the human 26-KD protein, interferon beta-2 (IFN-beta-2) as a B cell hybridoma/plasmocytoma growth factor induced by interleukin 1 and tumor necrosis factor. *J. Exp. Med.*, **165,** 914.

VILCEK, J., PALOMBELLA, V.J., HENRIKSEN-DE STEFANO, L. & 4 others (1986). Fibroblast growth enhancing activity of TNF and its relationship to other polypeptide growth factors. *J. Exp. Med.*, **163,** 632.

WAAGE, A., HALSTENSEN, A. & ESPEVIK, T. (1987). Association between tumour necrosis factor in serum and fatal outcome in patients with meningococcal disease. *Lancet*, **i,** 355.

WANG, A.M., CREASEY, A.A., LADNER, M.B. & 5 others (1985). Molecular cloning of the complementary DNA for human tumor necrosis factor. *Science*, **228,** 149.

WARREN, M.K. & RALPH, P. (1986). Macrophage growth factor CSF-1 stimulates human monocyte production of interferon, tumor necrosis factor, and colony stimulating activity. *J. Immunol.*, **137,** 2281.

WHITESIDE, T.L., WANG, Y.L. & HERBERMAN, R.B. (1989). Synergy between tumor necrosis factor alpha (TNF-α) and interleukin-2 (IL2): generation of CD8+ effectors from human tumor-infiltrating lymphocytes. *Proc. Am. Assoc. Cancer Res.*, **30,** 409.

WILLIAMSON, B.D., CARSWELL, E.A., RUBIN, B.J., PREDEGAST, J.S. & OLD, L.J. (1983). Human tumor necrosis factor produced by human B-cell lines: synergistic cytotoxic interaction with human interferon. *Proc. Natl Acad. Sci. USA*, **80,** 5397.

WONG, G.H.W. & GOEDDEL, D.V. (1986). Tumor necrosis factor alpha and beta inhibit virus replication and synergize with interferons. *Nature*, **323,** 819.

WRIGHT, S.C., JEWETT, A., MITSUYASU, R. & BONAVIDA, B. (1988). Spontaneous cytotoxicity and tumor necrosis factor production of peripheral blood monocytes from AIDS patients. *J. Immunol.*, **141,** 99.

YANG, S.C., OWEN-SHAUB, L., GRIMM, E.A. & ROTH, J.A. (1989). Induction of lymphokine-activated killer cytotoxicity with interleukin-2 and tumor necrosis factor-alpha against primary lung cancer targets. *Cancer Immunol. Immunother.*, **29,** 193.

YOUNG, J.D., LIU, C.C., CULTER, G., COHN, Z.A. & GALLI, S.J. (1987). Identification, purification, and characterization of a mast cell-association cytolytic factor related to tumor necrosis factor. *Proc. Natl Acad. Sci. USA*, **84,** 9175.

ZUCALI, J.R., BROXMEYER, H.E., GROSS, M.A. & DINARELLO, C.A. (1988). Recombinant tumor necrosis factor alpha and beta stimulate fibroblasts to produce hematopoietic growth factors in vitro. *J. Immunol.*, **140,** 840.

ZUCALI, J.R., ELFENBEIN, G.J., BARTH, K.C. & DINARELLO, C.A. (1987). Effects of human interleukin-1 and tumor necrosis factor on human T lymphocyte colony formation. *J. Clin. Invest.*, **80,** 772.


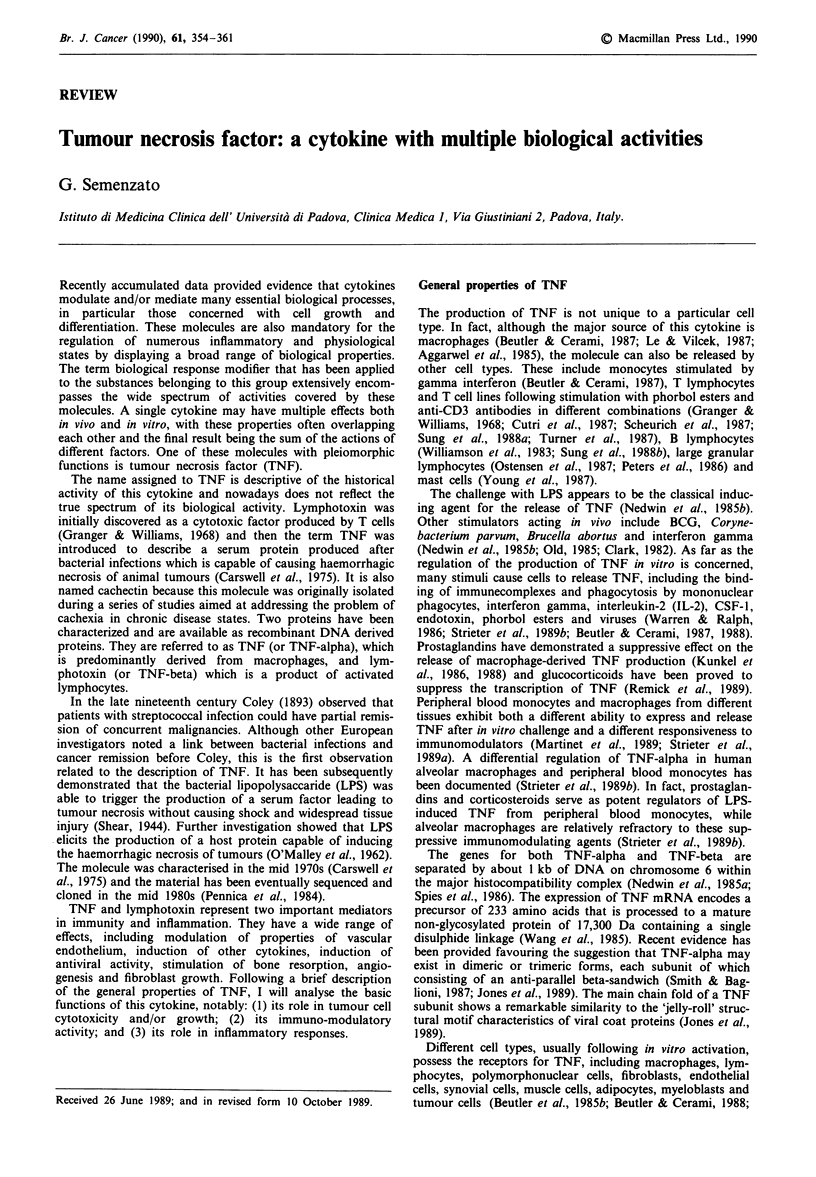

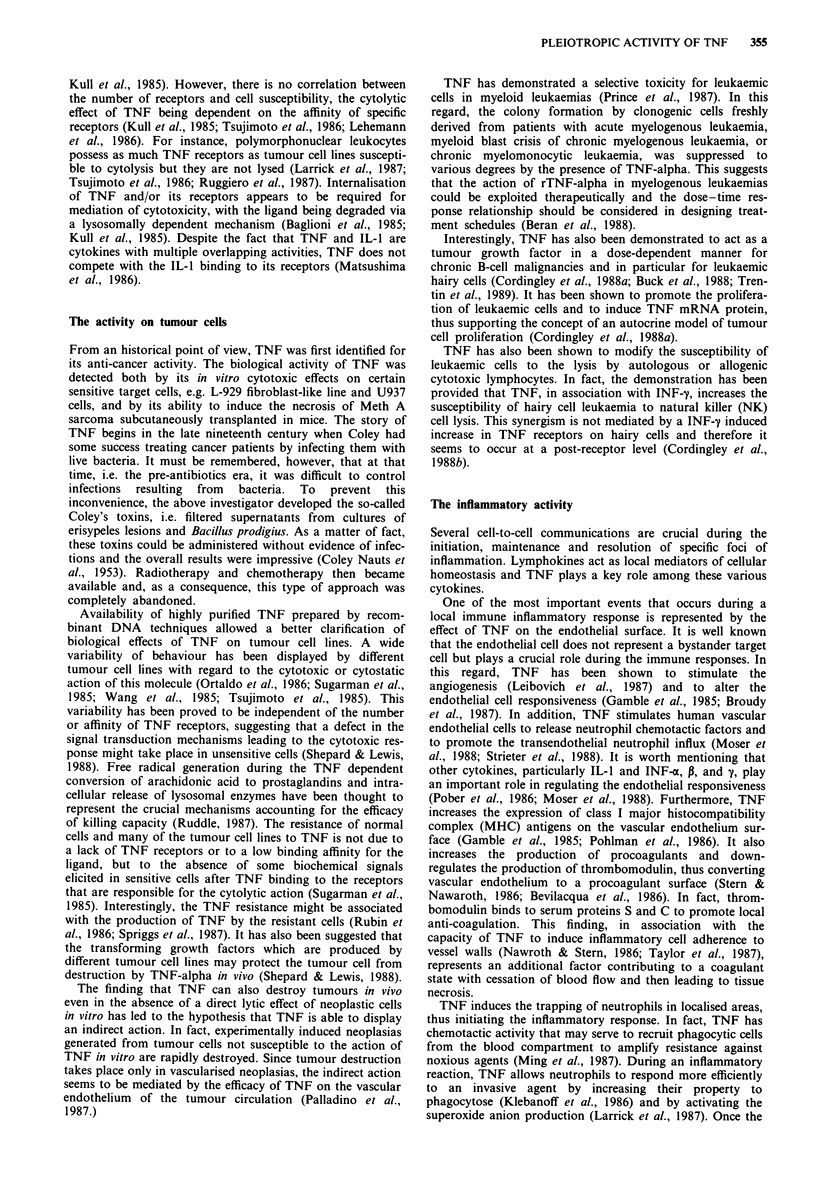

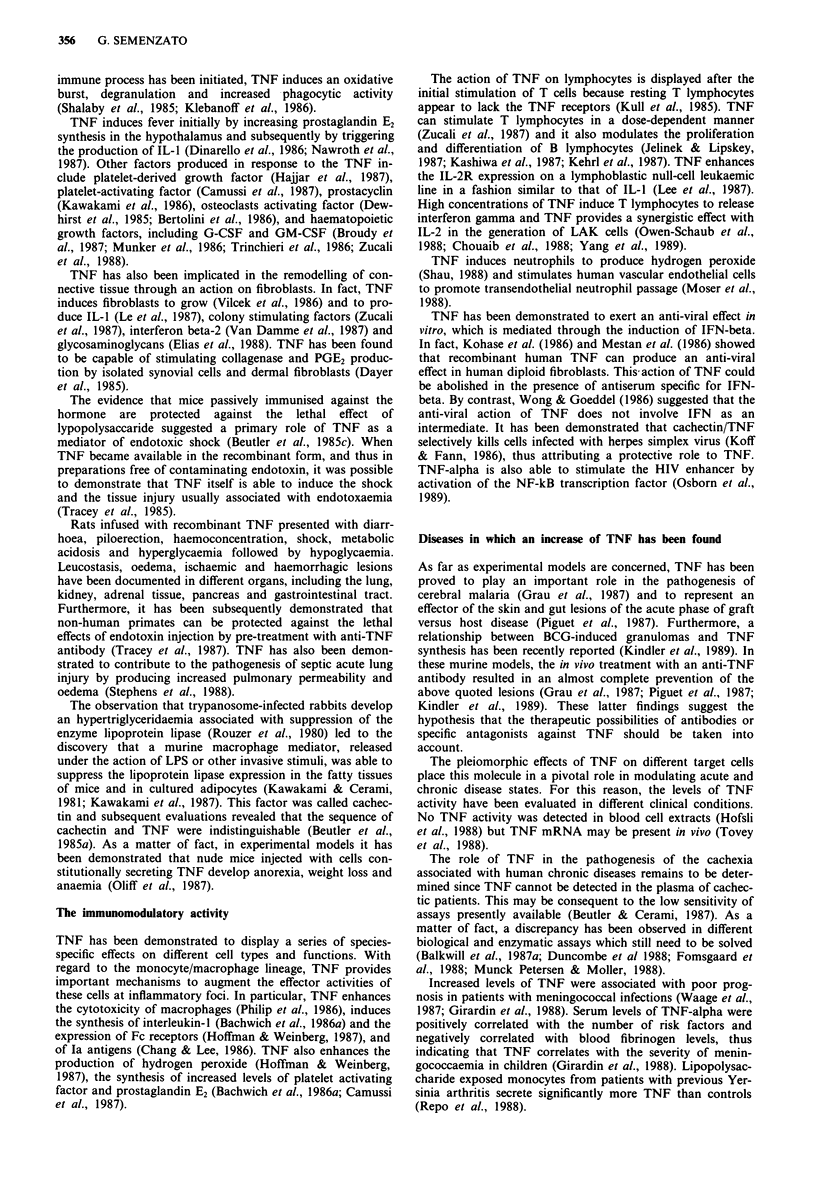

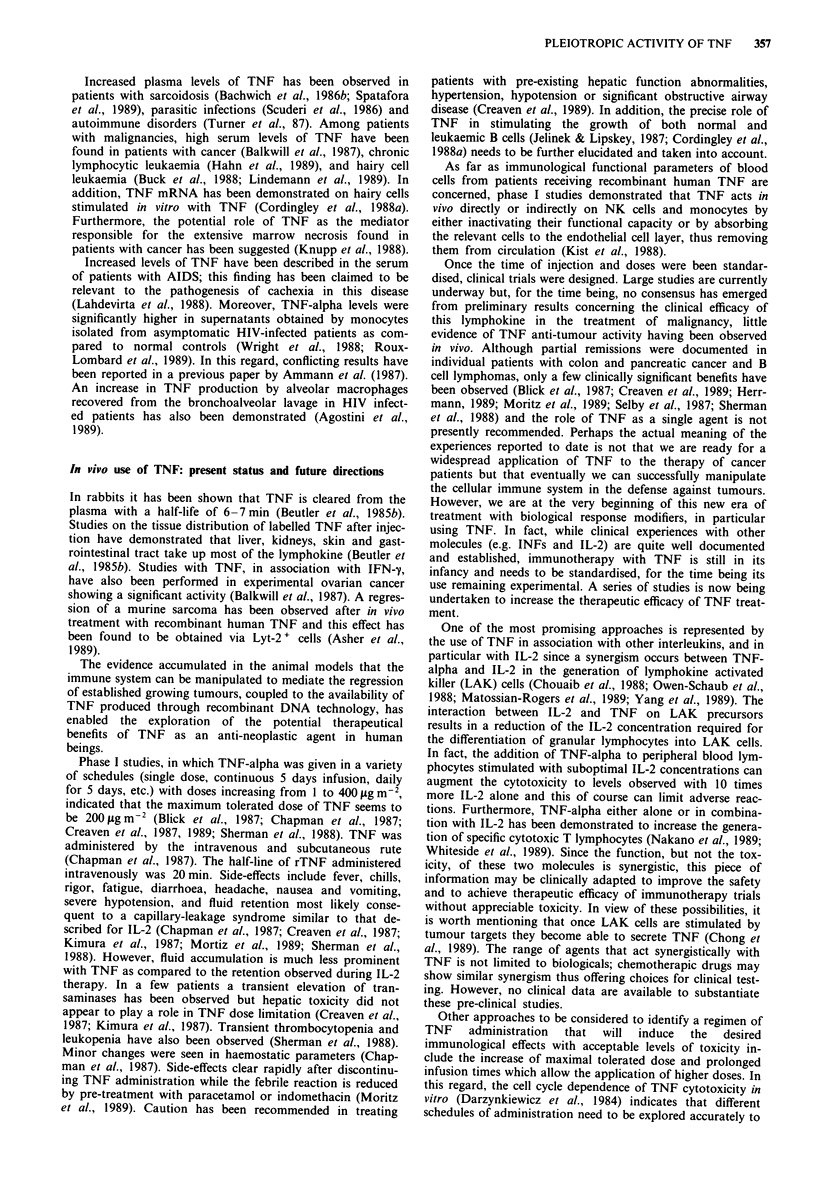

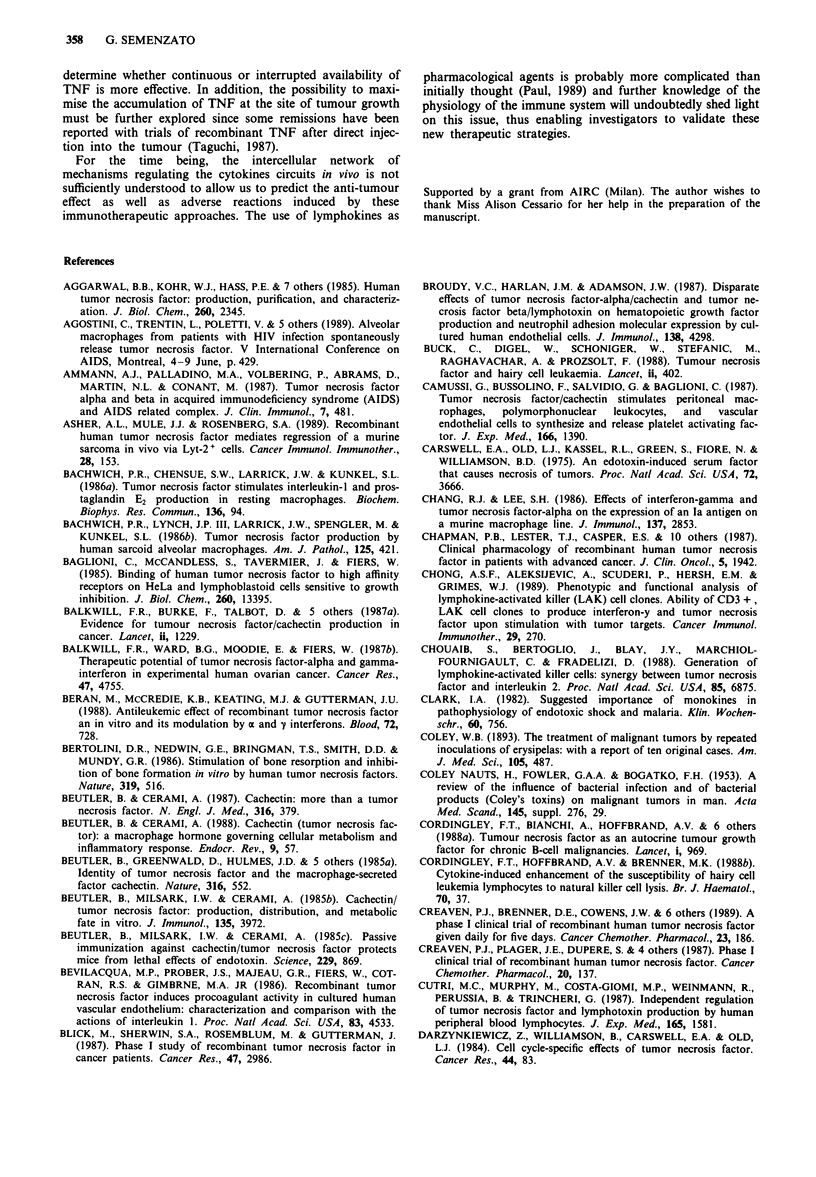

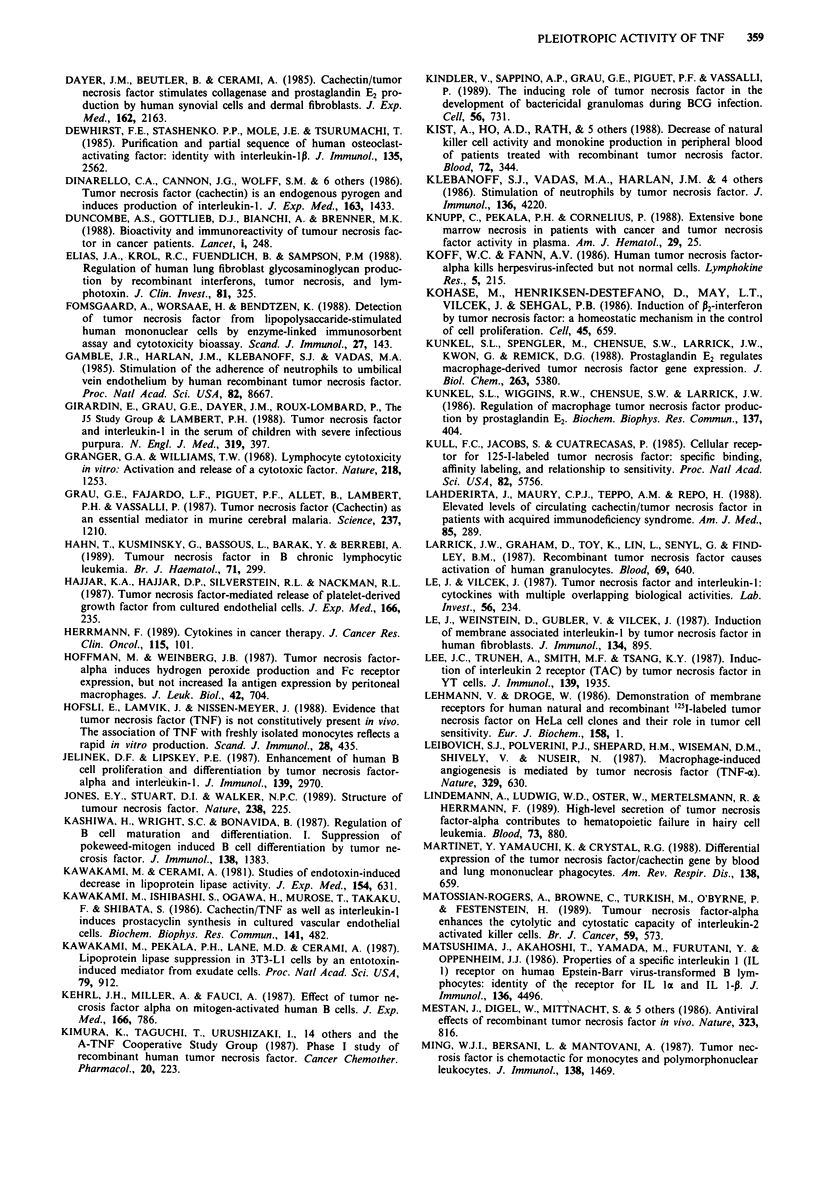

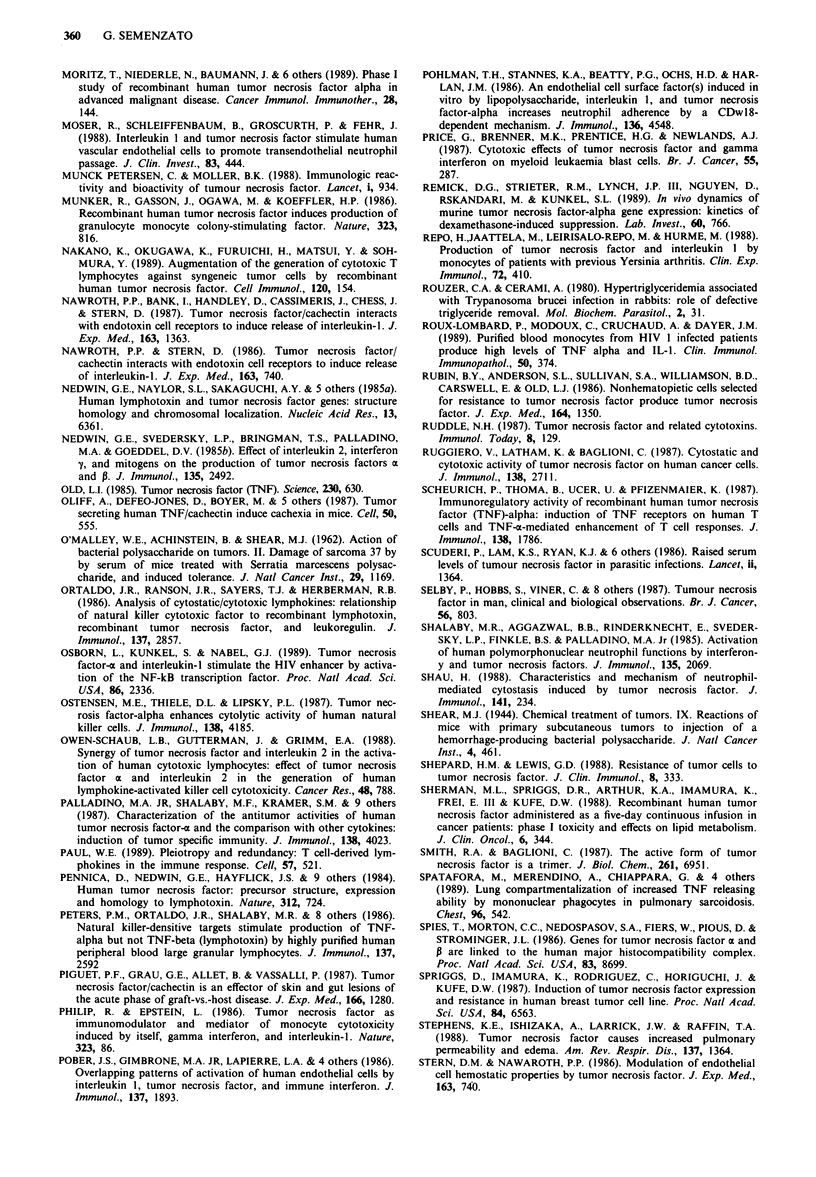

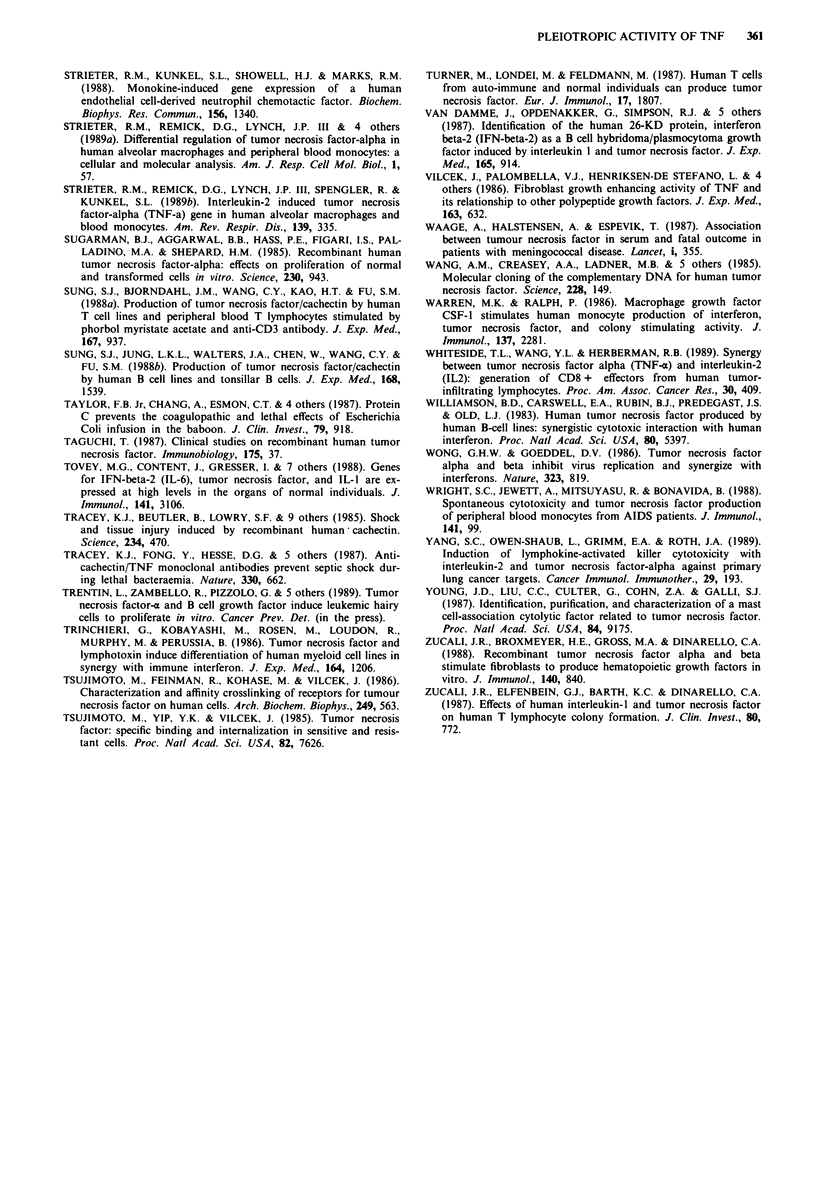


## References

[OCR_00656] Aggarwal B. B., Kohr W. J., Hass P. E., Moffat B., Spencer S. A., Henzel W. J., Bringman T. S., Nedwin G. E., Goeddel D. V., Harkins R. N. (1985). Human tumor necrosis factor. Production, purification, and characterization.. J Biol Chem.

[OCR_00667] Ammann A. J., Palladino M. A., Volberding P., Abrams D., Martin N. L., Conant M. (1987). Tumor necrosis factors alpha and beta in acquired immunodeficiency syndrome (AIDS) and aids-related complex.. J Clin Immunol.

[OCR_00673] Asher A. L., Mulé J. J., Rosenberg S. A. (1989). Recombinant human tumor necrosis factor mediates regression of a murine sarcoma in vivo via Lyt-2+ cells.. Cancer Immunol Immunother.

[OCR_00679] Bachwich P. R., Chensue S. W., Larrick J. W., Kunkel S. L. (1986). Tumor necrosis factor stimulates interleukin-1 and prostaglandin E2 production in resting macrophages.. Biochem Biophys Res Commun.

[OCR_00685] Bachwich P. R., Lynch J. P., Larrick J., Spengler M., Kunkel S. L. (1986). Tumor necrosis factor production by human sarcoid alveolar macrophages.. Am J Pathol.

[OCR_00689] Baglioni C., McCandless S., Tavernier J., Fiers W. (1985). Binding of human tumor necrosis factor to high affinity receptors on HeLa and lymphoblastoid cells sensitive to growth inhibition.. J Biol Chem.

[OCR_00700] Balkwill F. R., Ward B. G., Moodie E., Fiers W. (1987). Therapeutic potential of tumor necrosis factor-alpha and gamma-interferon in experimental human ovarian cancer.. Cancer Res.

[OCR_00706] Beran M., McCredie K. B., Keating M. J., Gutterman J. U. (1988). Antileukemic effect of recombinant tumor necrosis factor alpha in vitro and its modulation by alpha and gamma interferons.. Blood.

[OCR_00712] Bertolini D. R., Nedwin G. E., Bringman T. S., Smith D. D., Mundy G. R. (1986). Stimulation of bone resorption and inhibition of bone formation in vitro by human tumour necrosis factors.. Nature.

[OCR_00732] Beutler B. A., Milsark I. W., Cerami A. (1985). Cachectin/tumor necrosis factor: production, distribution, and metabolic fate in vivo.. J Immunol.

[OCR_00722] Beutler B., Cerami A. (1988). Cachectin (tumor necrosis factor): a macrophage hormone governing cellular metabolism and inflammatory response.. Endocr Rev.

[OCR_00718] Beutler B., Cerami A. (1987). Cachectin: more than a tumor necrosis factor.. N Engl J Med.

[OCR_00727] Beutler B., Greenwald D., Hulmes J. D., Chang M., Pan Y. C., Mathison J., Ulevitch R., Cerami A. (1985). Identity of tumour necrosis factor and the macrophage-secreted factor cachectin.. Nature.

[OCR_00737] Beutler B., Milsark I. W., Cerami A. C. (1985). Passive immunization against cachectin/tumor necrosis factor protects mice from lethal effect of endotoxin.. Science.

[OCR_00744] Bevilacqua M. P., Pober J. S., Majeau G. R., Fiers W., Cotran R. S., Gimbrone M. A. (1986). Recombinant tumor necrosis factor induces procoagulant activity in cultured human vascular endothelium: characterization and comparison with the actions of interleukin 1.. Proc Natl Acad Sci U S A.

[OCR_00748] Blick M., Sherwin S. A., Rosenblum M., Gutterman J. (1987). Phase I study of recombinant tumor necrosis factor in cancer patients.. Cancer Res.

[OCR_00753] Broudy V. C., Harlan J. M., Adamson J. W. (1987). Disparate effects of tumor necrosis factor-alpha/cachectin and tumor necrosis factor-beta/lymphotoxin on hematopoietic growth factor production and neutrophil adhesion molecule expression by cultured human endothelial cells.. J Immunol.

[OCR_00760] Buck C., Digel W., Schöniger W., Stefanic M., Raghavachar A., Porzsolt F. (1988). Tumour necrosis factor and hairy cell leukaemia.. Lancet.

[OCR_00765] Camussi G., Bussolino F., Salvidio G., Baglioni C. (1987). Tumor necrosis factor/cachectin stimulates peritoneal macrophages, polymorphonuclear neutrophils, and vascular endothelial cells to synthesize and release platelet-activating factor.. J Exp Med.

[OCR_00772] Carswell E. A., Old L. J., Kassel R. L., Green S., Fiore N., Williamson B. (1975). An endotoxin-induced serum factor that causes necrosis of tumors.. Proc Natl Acad Sci U S A.

[OCR_00778] Chang R. J., Lee S. H. (1986). Effects of interferon-gamma and tumor necrosis factor-alpha on the expression of an Ia antigen on a murine macrophage cell line.. J Immunol.

[OCR_00783] Chapman P. B., Lester T. J., Casper E. S., Gabrilove J. L., Wong G. Y., Kempin S. J., Gold P. J., Welt S., Warren R. S., Starnes H. F. (1987). Clinical pharmacology of recombinant human tumor necrosis factor in patients with advanced cancer.. J Clin Oncol.

[OCR_00787] Chong A. S., Aleksijevic A., Scuderi P., Hersh E. M., Grimes W. J. (1989). Phenotypic and functional analysis of lymphokine-activated killer (LAK) cell clones. Ability of CD3+, LAK cell clones to produce interferon-gamma and tumor necrosis factor upon stimulation with tumor targets.. Cancer Immunol Immunother.

[OCR_00797] Chouaib S., Bertoglio J., Blay J. Y., Marchiol-Fournigault C., Fradelizi D. (1988). Generation of lymphokine-activated killer cells: synergy between tumor necrosis factor and interleukin 2.. Proc Natl Acad Sci U S A.

[OCR_00800] Clark I. A. (1982). Suggested importance of monokines in pathophysiology of endotoxin shock and malaria.. Klin Wochenschr.

[OCR_00821] Cordingley F. T., Hoffbrand A. V., Brenner M. K. (1988). Cytokine-induced enhancement of the susceptibility of hairy cell leukaemia lymphocytes to natural killer cell lysis.. Br J Haematol.

[OCR_00827] Creaven P. J., Brenner D. E., Cowens J. W., Huben R. P., Wolf R. M., Takita H., Arbuck S. G., Razack M. S., Proefrock A. D. (1989). A phase I clinical trial of recombinant human tumor necrosis factor given daily for five days.. Cancer Chemother Pharmacol.

[OCR_00831] Creaven P. J., Plager J. E., Dupere S., Huben R. P., Takita H., Mittelman A., Proefrock A. (1987). Phase I clinical trial of recombinant human tumor necrosis factor.. Cancer Chemother Pharmacol.

[OCR_00836] Cuturi M. C., Murphy M., Costa-Giomi M. P., Weinmann R., Perussia B., Trinchieri G. (1987). Independent regulation of tumor necrosis factor and lymphotoxin production by human peripheral blood lymphocytes.. J Exp Med.

[OCR_00842] Darzynkiewicz Z., Williamson B., Carswell E. A., Old L. J. (1984). Cell cycle-specific effects of tumor necrosis factor.. Cancer Res.

[OCR_00849] Dayer J. M., Beutler B., Cerami A. (1985). Cachectin/tumor necrosis factor stimulates collagenase and prostaglandin E2 production by human synovial cells and dermal fibroblasts.. J Exp Med.

[OCR_00855] Dewhirst F. E., Stashenko P. P., Mole J. E., Tsurumachi T. (1985). Purification and partial sequence of human osteoclast-activating factor: identity with interleukin 1 beta.. J Immunol.

[OCR_00861] Dinarello C. A., Cannon J. G., Wolff S. M., Bernheim H. A., Beutler B., Cerami A., Figari I. S., Palladino M. A., O'Connor J. V. (1986). Tumor necrosis factor (cachectin) is an endogenous pyrogen and induces production of interleukin 1.. J Exp Med.

[OCR_00866] Duncombe A. S., Gottlieb D. J., Bianchi A., Brenner M. K. (1988). Bioactivity and immunoreactivity of tumour necrosis factor in cancer patients.. Lancet.

[OCR_00871] Elias J. A., Krol R. C., Freundlich B., Sampson P. M. (1988). Regulation of human lung fibroblast glycosaminoglycan production by recombinant interferons, tumor necrosis factor, and lymphotoxin.. J Clin Invest.

[OCR_00877] Fomsgaard A., Worsaae H., Bendtzen K. (1988). Detection of tumour necrosis factor from lipopolysaccharide-stimulated human mononuclear cells by enzyme-linked immunosorbent assay and cytotoxicity bioassay.. Scand J Immunol.

[OCR_00883] Gamble J. R., Harlan J. M., Klebanoff S. J., Vadas M. A. (1985). Stimulation of the adherence of neutrophils to umbilical vein endothelium by human recombinant tumor necrosis factor.. Proc Natl Acad Sci U S A.

[OCR_00889] Girardin E., Grau G. E., Dayer J. M., Roux-Lombard P., Lambert P. H. (1988). Tumor necrosis factor and interleukin-1 in the serum of children with severe infectious purpura.. N Engl J Med.

[OCR_00895] Granger G. A., Williams T. W. (1968). Lymphocyte cytotoxicity in vitro: activation and release of a cytotoxic factor.. Nature.

[OCR_00900] Grau G. E., Fajardo L. F., Piguet P. F., Allet B., Lambert P. H., Vassalli P. (1987). Tumor necrosis factor (cachectin) as an essential mediator in murine cerebral malaria.. Science.

[OCR_00906] Hahn T., Kusminsky G., Bassous L., Barak Y., Berrebi A. (1989). Tumour necrosis factor in B chronic lymphocytic leukaemia.. Br J Haematol.

[OCR_00911] Hajjar K. A., Hajjar D. P., Silverstein R. L., Nachman R. L. (1987). Tumor necrosis factor-mediated release of platelet-derived growth factor from cultured endothelial cells.. J Exp Med.

[OCR_00921] Hoffman M., Weinberg J. B. (1987). Tumor necrosis factor-alpha induces increased hydrogen peroxide production and Fc receptor expression, but not increased Ia antigen expression by peritoneal macrophages.. J Leukoc Biol.

[OCR_00927] Hofsli E., Lamvik J., Nissen-Meyer J. (1988). Evidence that tumour necrosis factor (TNF) is not constitutively present in vivo. The association of TNF with freshly isolated monocytes reflects a rapid in vitro production.. Scand J Immunol.

[OCR_00933] Jelinek D. F., Lipsky P. E. (1987). Enhancement of human B cell proliferation and differentiation by tumor necrosis factor-alpha and interleukin 1.. J Immunol.

[OCR_00938] Jones E. Y., Stuart D. I., Walker N. P. (1989). Structure of tumour necrosis factor.. Nature.

[OCR_00942] Kashiwa H., Wright S. C., Bonavida B. (1987). Regulation of B cell maturation and differentiation. I. Suppression of pokeweed mitogen-induced B cell differentiation by tumor necrosis factor (TNF).. J Immunol.

[OCR_00948] Kawakami M., Cerami A. (1981). Studies of endotoxin-induced decrease in lipoprotein lipase activity.. J Exp Med.

[OCR_00952] Kawakami M., Ishibashi S., Ogawa H., Murase T., Takaku F., Shibata S. (1986). Cachectin/TNF as well as interleukin-1 induces prostacyclin synthesis in cultured vascular endothelial cells.. Biochem Biophys Res Commun.

[OCR_00958] Kawakami M., Pekala P. H., Lane M. D., Cerami A. (1982). Lipoprotein lipase suppression in 3T3-L1 cells by an endotoxin-induced mediator from exudate cells.. Proc Natl Acad Sci U S A.

[OCR_00964] Kehrl J. H., Miller A., Fauci A. S. (1987). Effect of tumor necrosis factor alpha on mitogen-activated human B cells.. J Exp Med.

[OCR_00969] Kimura K., Taguchi T., Urushizaki I., Ohno R., Abe O., Furue H., Hattori T., Ichihashi H., Inoguchi K., Majima H. (1987). Phase I study of recombinant human tumor necrosis factor.. Cancer Chemother Pharmacol.

[OCR_00975] Kindler V., Sappino A. P., Grau G. E., Piguet P. F., Vassalli P. (1989). The inducing role of tumor necrosis factor in the development of bactericidal granulomas during BCG infection.. Cell.

[OCR_00981] Kist A., Ho A. D., Räth U., Wiedenmann B., Bauer A., Schlick E., Kirchner H., Männel D. N. (1988). Decrease of natural killer cell activity and monokine production in peripheral blood of patients treated with recombinant tumor necrosis factor.. Blood.

[OCR_00987] Klebanoff S. J., Vadas M. A., Harlan J. M., Sparks L. H., Gamble J. R., Agosti J. M., Waltersdorph A. M. (1986). Stimulation of neutrophils by tumor necrosis factor.. J Immunol.

[OCR_00997] Koff W. C., Fann A. V. (1986). Human tumor necrosis factor-alpha kills herpesvirus-infected but not normal cells.. Lymphokine Res.

[OCR_01002] Kohase M., Henriksen-DeStefano D., May L. T., Vilcek J., Sehgal P. B. (1986). Induction of beta 2-interferon by tumor necrosis factor: a homeostatic mechanism in the control of cell proliferation.. Cell.

[OCR_01020] Kull F. C., Jacobs S., Cuatrecasas P. (1985). Cellular receptor for 125I-labeled tumor necrosis factor: specific binding, affinity labeling, and relationship to sensitivity.. Proc Natl Acad Sci U S A.

[OCR_01008] Kunkel S. L., Spengler M., May M. A., Spengler R., Larrick J., Remick D. (1988). Prostaglandin E2 regulates macrophage-derived tumor necrosis factor gene expression.. J Biol Chem.

[OCR_01014] Kunkel S. L., Wiggins R. C., Chensue S. W., Larrick J. (1986). Regulation of macrophage tumor necrosis factor production by prostaglandin E2.. Biochem Biophys Res Commun.

[OCR_01032] Larrick J. W., Graham D., Toy K., Lin L. S., Senyk G., Fendly B. M. (1987). Recombinant tumor necrosis factor causes activation of human granulocytes.. Blood.

[OCR_01037] Le J., Vilcek J. (1987). Tumor necrosis factor and interleukin 1: cytokines with multiple overlapping biological activities.. Lab Invest.

[OCR_01047] Lee J. C., Truneh A., Smith M. F., Tsang K. Y. (1987). Induction of interleukin 2 receptor (TAC) by tumor necrosis factor in YT cells.. J Immunol.

[OCR_01052] Lehmann V., Dröge W. (1986). Demonstration of membrane receptors for human natural and recombinant 125I-labeled tumor necrosis factor on HeLa cell clones and their role in tumor cell sensitivity.. Eur J Biochem.

[OCR_01058] Leibovich S. J., Polverini P. J., Shepard H. M., Wiseman D. M., Shively V., Nuseir N. (1987). Macrophage-induced angiogenesis is mediated by tumour necrosis factor-alpha.. Nature.

[OCR_01064] Lindemann A., Ludwig W. D., Oster W., Mertelsmann R., Herrmann F. (1989). High-level secretion of tumor necrosis factor-alpha contributes to hematopoietic failure in hairy cell leukemia.. Blood.

[OCR_01026] Lähdevirta J., Maury C. P., Teppo A. M., Repo H. (1988). Elevated levels of circulating cachectin/tumor necrosis factor in patients with acquired immunodeficiency syndrome.. Am J Med.

[OCR_01070] Martinet Y., Yamauchi K., Crystal R. G. (1988). Differential expression of the tumor necrosis factor/cachectin gene by blood and lung mononuclear phagocytes.. Am Rev Respir Dis.

[OCR_01076] Matossian-Rogers A., Browne C., Turkish M., O'Byrne P., Festenstein H. (1989). Tumour necrosis factor-alpha enhances the cytolytic and cytostatic capacity of interleukin-2 activated killer cells.. Br J Cancer.

[OCR_01082] Matsushima K., Akahoshi T., Yamada M., Furutani Y., Oppenheim J. J. (1986). Properties of a specific interleukin 1 (IL 1) receptor on human Epstein Barr virus-transformed B lymphocytes: identity of the receptor for IL 1-alpha and IL 1-beta.. J Immunol.

[OCR_01089] Mestan J., Digel W., Mittnacht S., Hillen H., Blohm D., Möller A., Jacobsen H., Kirchner H. Antiviral effects of recombinant tumour necrosis factor in vitro.. Nature.

[OCR_01116] Mestan J., Digel W., Mittnacht S., Hillen H., Blohm D., Möller A., Jacobsen H., Kirchner H. Antiviral effects of recombinant tumour necrosis factor in vitro.. Nature.

[OCR_01094] Ming W. J., Bersani L., Mantovani A. (1987). Tumor necrosis factor is chemotactic for monocytes and polymorphonuclear leukocytes.. J Immunol.

[OCR_01101] Moritz T., Niederle N., Baumann J., May D., Kurschel E., Osieka R., Kempeni J., Schlick E., Schmidt C. G. (1989). Phase I study of recombinant human tumor necrosis factor alpha in advanced malignant disease.. Cancer Immunol Immunother.

[OCR_01107] Moser R., Schleiffenbaum B., Groscurth P., Fehr J. (1989). Interleukin 1 and tumor necrosis factor stimulate human vascular endothelial cells to promote transendothelial neutrophil passage.. J Clin Invest.

[OCR_01124] Nakano K., Okugawa K., Furuichi H., Matsui Y., Sohmura Y. (1989). Augmentation of the generation of cytotoxic T lymphocytes against syngeneic tumor cells by recombinant human tumor necrosis factor.. Cell Immunol.

[OCR_01128] Nawroth P. P., Bank I., Handley D., Cassimeris J., Chess L., Stern D. (1986). Tumor necrosis factor/cachectin interacts with endothelial cell receptors to induce release of interleukin 1.. J Exp Med.

[OCR_01348] Nawroth P. P., Stern D. M. (1986). Modulation of endothelial cell hemostatic properties by tumor necrosis factor.. J Exp Med.

[OCR_01134] Nawroth P. P., Stern D. M. (1986). Modulation of endothelial cell hemostatic properties by tumor necrosis factor.. J Exp Med.

[OCR_01139] Nedwin G. E., Naylor S. L., Sakaguchi A. Y., Smith D., Jarrett-Nedwin J., Pennica D., Goeddel D. V., Gray P. W. (1985). Human lymphotoxin and tumor necrosis factor genes: structure, homology and chromosomal localization.. Nucleic Acids Res.

[OCR_01145] Nedwin G. E., Svedersky L. P., Bringman T. S., Palladino M. A., Goeddel D. V. (1985). Effect of interleukin 2, interferon-gamma, and mitogens on the production of tumor necrosis factors alpha and beta.. J Immunol.

[OCR_01151] Old L. J. (1985). Tumor necrosis factor (TNF).. Science.

[OCR_01153] Oliff A., Defeo-Jones D., Boyer M., Martinez D., Kiefer D., Vuocolo G., Wolfe A., Socher S. H. (1987). Tumors secreting human TNF/cachectin induce cachexia in mice.. Cell.

[OCR_01163] Ortaldo J. R., Ransom J. R., Sayers T. J., Herberman R. B. (1986). Analysis of cytostatic/cytotoxic lymphokines: relationship of natural killer cytotoxic factor to recombinant lymphotoxin, recombinant tumor necrosis factor, and leukoregulin.. J Immunol.

[OCR_01170] Osborn L., Kunkel S., Nabel G. J. (1989). Tumor necrosis factor alpha and interleukin 1 stimulate the human immunodeficiency virus enhancer by activation of the nuclear factor kappa B.. Proc Natl Acad Sci U S A.

[OCR_01176] Ostensen M. E., Thiele D. L., Lipsky P. E. (1987). Tumor necrosis factor-alpha enhances cytolytic activity of human natural killer cells.. J Immunol.

[OCR_01181] Owen-Schaub L. B., Gutterman J. U., Grimm E. A. (1988). Synergy of tumor necrosis factor and interleukin 2 in the activation of human cytotoxic lymphocytes: effect of tumor necrosis factor alpha and interleukin 2 in the generation of human lymphokine-activated killer cell cytotoxicity.. Cancer Res.

[OCR_01187] Palladino M. A., Shalaby M. R., Kramer S. M., Ferraiolo B. L., Baughman R. A., Deleo A. B., Crase D., Marafino B., Aggarwal B. B., Figari I. S. (1987). Characterization of the antitumor activities of human tumor necrosis factor-alpha and the comparison with other cytokines: induction of tumor-specific immunity.. J Immunol.

[OCR_01193] Paul W. E. (1989). Pleiotropy and redundancy: T cell-derived lymphokines in the immune response.. Cell.

[OCR_01199] Pennica D., Nedwin G. E., Hayflick J. S., Seeburg P. H., Derynck R., Palladino M. A., Kohr W. J., Aggarwal B. B., Goeddel D. V. (1984). Human tumour necrosis factor: precursor structure, expression and homology to lymphotoxin.. Nature.

[OCR_01202] Peters P. M., Ortaldo J. R., Shalaby M. R., Svedersky L. P., Nedwin G. E., Bringman T. S., Hass P. E., Aggarwal B. B., Herberman R. B., Goeddel D. V. (1986). Natural killer-sensitive targets stimulate production of TNF-alpha but not TNF-beta (lymphotoxin) by highly purified human peripheral blood large granular lymphocytes.. J Immunol.

[OCR_01113] Petersen C. M., Møller B. K. (1988). Immunological reactivity and bioactivity of tumour necrosis factor.. Lancet.

[OCR_01213] Philip R., Epstein L. B. (1986). Tumour necrosis factor as immunomodulator and mediator of monocyte cytotoxicity induced by itself, gamma-interferon and interleukin-1.. Nature.

[OCR_01209] Piguet P. F., Grau G. E., Allet B., Vassalli P. (1987). Tumor necrosis factor/cachectin is an effector of skin and gut lesions of the acute phase of graft-vs.-host disease.. J Exp Med.

[OCR_01219] Pober J. S., Gimbrone M. A., Lapierre L. A., Mendrick D. L., Fiers W., Rothlein R., Springer T. A. (1986). Overlapping patterns of activation of human endothelial cells by interleukin 1, tumor necrosis factor, and immune interferon.. J Immunol.

[OCR_01227] Pohlman T. H., Stanness K. A., Beatty P. G., Ochs H. D., Harlan J. M. (1986). An endothelial cell surface factor(s) induced in vitro by lipopolysaccharide, interleukin 1, and tumor necrosis factor-alpha increases neutrophil adherence by a CDw18-dependent mechanism.. J Immunol.

[OCR_01232] Price G., Brenner M. K., Prentice H. G., Hoffbrand A. V., Newland A. C. (1987). Cytotoxic effects of tumour necrosis factor and gamma-interferon on acute myeloid leukaemia blasts.. Br J Cancer.

[OCR_01238] Remick D. G., Strieter R. M., Lynch J. P., Nguyen D., Eskandari M., Kunkel S. L. (1989). In vivo dynamics of murine tumor necrosis factor-alpha gene expression. Kinetics of dexamethasone-induced suppression.. Lab Invest.

[OCR_01244] Repo H., Jättelä M., Leirisalo-Repo M., Hurme M. (1988). Production of tumour necrosis factor and interleukin 1 by monocytes of patients with previous Yersinia arthritis.. Clin Exp Immunol.

[OCR_01255] Roux-Lombard P., Modoux C., Cruchaud A., Dayer J. M. (1989). Purified blood monocytes from HIV 1-infected patients produce high levels of TNF alpha and IL-1.. Clin Immunol Immunopathol.

[OCR_01250] Rouzer C. A., Cerami A. (1980). Hypertriglyceridemia associated with Trypanosoma brucei brucei infection in rabbits: role of defective triglyceride removal.. Mol Biochem Parasitol.

[OCR_01261] Rubin B. Y., Anderson S. L., Sullivan S. A., Williamson B. D., Carswell E. A., Old L. J. (1986). Nonhematopoietic cells selected for resistance to tumor necrosis factor produce tumor necrosis factor.. J Exp Med.

[OCR_01271] Ruggiero V., Latham K., Baglioni C. (1987). Cytostatic and cytotoxic activity of tumor necrosis factor on human cancer cells.. J Immunol.

[OCR_01276] Scheurich P., Thoma B., Ucer U., Pfizenmaier K. (1987). Immunoregulatory activity of recombinant human tumor necrosis factor (TNF)-alpha: induction of TNF receptors on human T cells and TNF-alpha-mediated enhancement of T cell responses.. J Immunol.

[OCR_01283] Scuderi P., Sterling K. E., Lam K. S., Finley P. R., Ryan K. J., Ray C. G., Petersen E., Slymen D. J., Salmon S. E. (1986). Raised serum levels of tumour necrosis factor in parasitic infections.. Lancet.

[OCR_01288] Selby P., Hobbs S., Viner C., Jackson E., Jones A., Newell D., Calvert A. H., McElwain T., Fearon K., Humphreys J. (1987). Tumour necrosis factor in man: clinical and biological observations.. Br J Cancer.

[OCR_01295] Shalaby M. R., Aggarwal B. B., Rinderknecht E., Svedersky L. P., Finkle B. S., Palladino M. A. (1985). Activation of human polymorphonuclear neutrophil functions by interferon-gamma and tumor necrosis factors.. J Immunol.

[OCR_01299] Shau H. (1988). Characteristics and mechanism of neutrophil-mediated cytostasis induced by tumor necrosis factor.. J Immunol.

[OCR_01310] Shepard H. M., Lewis G. D. (1988). Resistance of tumor cells to tumor necrosis factor.. J Clin Immunol.

[OCR_01314] Sherman M. L., Spriggs D. R., Arthur K. A., Imamura K., Frei E., Kufe D. W. (1988). Recombinant human tumor necrosis factor administered as a five-day continuous infusion in cancer patients: phase I toxicity and effects on lipid metabolism.. J Clin Oncol.

[OCR_01321] Smith R. A., Baglioni C. (1987). The active form of tumor necrosis factor is a trimer.. J Biol Chem.

[OCR_01325] Spatafora M., Merendino A., Chiappara G., Gjomarkaj M., Melis M., Bellia V., Bonsignore G. (1989). Lung compartmentalization of increased TNF releasing ability by mononuclear phagocytes in pulmonary sarcoidosis.. Chest.

[OCR_01331] Spies T., Morton C. C., Nedospasov S. A., Fiers W., Pious D., Strominger J. L. (1986). Genes for the tumor necrosis factors alpha and beta are linked to the human major histocompatibility complex.. Proc Natl Acad Sci U S A.

[OCR_01337] Spriggs D., Imamura K., Rodriguez C., Horiguchi J., Kufe D. W. (1987). Induction of tumor necrosis factor expression and resistance in a human breast tumor cell line.. Proc Natl Acad Sci U S A.

[OCR_01343] Stephens K. E., Ishizaka A., Larrick J. W., Raffin T. A. (1988). Tumor necrosis factor causes increased pulmonary permeability and edema. Comparison to septic acute lung injury.. Am Rev Respir Dis.

[OCR_01355] Strieter R. M., Kunkel S. L., Showell H. J., Marks R. M. (1988). Monokine-induced gene expression of a human endothelial cell-derived neutrophil chemotactic factor.. Biochem Biophys Res Commun.

[OCR_01376] Sugarman B. J., Aggarwal B. B., Hass P. E., Figari I. S., Palladino M. A., Shepard H. M. (1985). Recombinant human tumor necrosis factor-alpha: effects on proliferation of normal and transformed cells in vitro.. Science.

[OCR_01380] Sung S. S., Bjorndahl J. M., Wang C. Y., Kao H. T., Fu S. M. (1988). Production of tumor necrosis factor/cachectin by human T cell lines and peripheral blood T lymphocytes stimulated by phorbol myristate acetate and anti-CD3 antibody.. J Exp Med.

[OCR_01387] Sung S. S., Jung L. K., Walters J. A., Chen W., Wang C. Y., Fu S. M. (1988). Production of tumor necrosis factor/cachectin by human B cell lines and tonsillar B cells.. J Exp Med.

[OCR_01393] Taylor F. B., Chang A., Esmon C. T., D'Angelo A., Vigano-D'Angelo S., Blick K. E. (1987). Protein C prevents the coagulopathic and lethal effects of Escherichia coli infusion in the baboon.. J Clin Invest.

[OCR_01402] Tovey M. G., Content J., Gresser I., Gugenheim J., Blanchard B., Guymarho J., Poupart P., Gigou M., Shaw A., Fiers W. (1988). Genes for IFN-beta-2 (IL-6), tumor necrosis factor, and IL-1 are expressed at high levels in the organs of normal individuals.. J Immunol.

[OCR_01408] Tracey K. J., Beutler B., Lowry S. F., Merryweather J., Wolpe S., Milsark I. W., Hariri R. J., Fahey T. J., Zentella A., Albert J. D. (1986). Shock and tissue injury induced by recombinant human cachectin.. Science.

[OCR_01413] Tracey K. J., Fong Y., Hesse D. G., Manogue K. R., Lee A. T., Kuo G. C., Lowry S. F., Cerami A. (1987). Anti-cachectin/TNF monoclonal antibodies prevent septic shock during lethal bacteraemia.. Nature.

[OCR_01423] Trinchieri G., Kobayashi M., Rosen M., Loudon R., Murphy M., Perussia B. (1986). Tumor necrosis factor and lymphotoxin induce differentiation of human myeloid cell lines in synergy with immune interferon.. J Exp Med.

[OCR_01429] Tsujimoto M., Feinman R., Kohase M., Vilcek J. (1986). Characterization and affinity crosslinking of receptors for tumor necrosis factor on human cells.. Arch Biochem Biophys.

[OCR_01433] Tsujimoto M., Yip Y. K., Vilcek J. (1985). Tumor necrosis factor: specific binding and internalization in sensitive and resistant cells.. Proc Natl Acad Sci U S A.

[OCR_01438] Turner M., Londei M., Feldmann M. (1987). Human T cells from autoimmune and normal individuals can produce tumor necrosis factor.. Eur J Immunol.

[OCR_01443] Van Damme J., Opdenakker G., Simpson R. J., Rubira M. R., Cayphas S., Vink A., Billiau A., Van Snick J. (1987). Identification of the human 26-kD protein, interferon beta 2 (IFN-beta 2), as a B cell hybridoma/plasmacytoma growth factor induced by interleukin 1 and tumor necrosis factor.. J Exp Med.

[OCR_01450] Vilcek J., Palombella V. J., Henriksen-DeStefano D., Swenson C., Feinman R., Hirai M., Tsujimoto M. (1986). Fibroblast growth enhancing activity of tumor necrosis factor and its relationship to other polypeptide growth factors.. J Exp Med.

[OCR_01456] Waage A., Halstensen A., Espevik T. (1987). Association between tumour necrosis factor in serum and fatal outcome in patients with meningococcal disease.. Lancet.

[OCR_01461] Wang A. M., Creasey A. A., Ladner M. B., Lin L. S., Strickler J., Van Arsdell J. N., Yamamoto R., Mark D. F. (1985). Molecular cloning of the complementary DNA for human tumor necrosis factor.. Science.

[OCR_01466] Warren M. K., Ralph P. (1986). Macrophage growth factor CSF-1 stimulates human monocyte production of interferon, tumor necrosis factor, and colony stimulating activity.. J Immunol.

[OCR_01477] Williamson B. D., Carswell E. A., Rubin B. Y., Prendergast J. S., Old L. J. (1983). Human tumor necrosis factor produced by human B-cell lines: synergistic cytotoxic interaction with human interferon.. Proc Natl Acad Sci U S A.

[OCR_01483] Wong G. H., Goeddel D. V. Tumour necrosis factors alpha and beta inhibit virus replication and synergize with interferons.. Nature.

[OCR_01488] Wright S. C., Jewett A., Mitsuyasu R., Bonavida B. (1988). Spontaneous cytotoxicity and tumor necrosis factor production by peripheral blood monocytes from AIDS patients.. J Immunol.

[OCR_01494] Yang S. C., Owen-Schaub L., Grimm E. A., Roth J. A. (1989). Induction of lymphokine-activated killer cytotoxicity with interleukin-2 and tumor necrosis factor-alpha against primary lung cancer targets.. Cancer Immunol Immunother.

[OCR_01500] Young J. D., Liu C. C., Butler G., Cohn Z. A., Galli S. J. (1987). Identification, purification, and characterization of a mast cell-associated cytolytic factor related to tumor necrosis factor.. Proc Natl Acad Sci U S A.

[OCR_01506] Zucali J. R., Broxmeyer H. E., Gross M. A., Dinarello C. A. (1988). Recombinant human tumor necrosis factors alpha and beta stimulate fibroblasts to produce hemopoietic growth factors in vitro.. J Immunol.

[OCR_01512] Zucali J. R., Elfenbein G. J., Barth K. C., Dinarello C. A. (1987). Effects of human interleukin 1 and human tumor necrosis factor on human T lymphocyte colony formation.. J Clin Invest.

